# ER stress regulating protein phosphatase 2A-B56γ, targeted by hepatitis B virus X protein, induces cell cycle arrest and apoptosis of hepatocytes

**DOI:** 10.1038/s41419-018-0787-3

**Published:** 2018-07-09

**Authors:** Chengyong He, Yang Qiu, Peiyu Han, Yuanyuan Chen, Liyin Zhang, Quan Yuan, Tianying Zhang, Tong Cheng, Lunzhi Yuan, Chenghao Huang, Sheng Zhang, Zhenyu Yin, Xian-E. Peng, Dong Liang, Xu Lin, Yuchun Lin, Zhongning Lin, Ningshao Xia

**Affiliations:** 1 State Key Laboratory of Molecular Vaccinology and Molecular Diagnostics, School of Public Health, Xiamen University, Xiamen, China; 2 Department of Hepatobiliary Surgery, Fujian Provincial Key Laboratory of Chronic Liver Disease and Hepatocellular Carcinoma, Xiamen University Affiliated Zhongshan Hospital, Xiamen, China; 3 Key Laboratory of Ministry of Education for Gastrointestinal Cancer, School of Basic Medical Sciences, Fujian Medical University, Fuzhou, China; 4 Mengchao Hepatobiliary Hospital of Fujian Medical University, Fuzhou, China

## Abstract

Hepatitis B virus X (HBx) protein contributes to the progression of hepatitis B virus (HBV)-related hepatic injury and diseases, but the exact mechanism remains unclear. Protein phosphatase 2 A (PP2A) is a major serine/threonine phosphatase involved in regulating many cellular phosphorylation signals that are important for regulation of cell cycle and apoptosis. Does HBx target to PP2A-B56γ and therefore affect HBx-induced hepatotoxicity? In the present study, the expression of B56γ positively correlated with the level of HBx in HBV-infected primary human hepatocytes in human-liver-chimeric mice, HBx-transgenic mice, HBV-infected cells, and HBx-expressing hepatic cells. B56γ promoted p53/p21-dependent cell cycle arrest and apoptosis. Mechanistically, B56γ was transactivated by AP-1, which was under the regulation of endoplasmic reticulum (ER) stress induced CREBH signaling in HBx-expressing hepatic cells. B56γ dephosphorylated p-Thr55-p53 to trigger p53/p21 pathway-dependent cell cycle G1 phase arrest, resulting in apoptosis of hepatic cells. In conclusion, this study provides a novel insight into a mechanism of B56γ mediating cell cycle arrest and apoptosis of HBx-expressing hepatic cells and a basis for B56γ being a potential therapeutic target for HBV-infected hepatic cells.

## Introduction

Hepatitis B virus (HBV), chronically infecting estimated 257 million people worldwide, is one of the most important etiological factors causing hepatitis and hepatic injury^1^. Chronic HBV infection leads to progressive complications via several molecular mechanisms and cellular signaling pathways^2^. Although the exact mechanisms by which chronic HBV infection leads to hepatic injury are still unclear, HBV proteins are thought to play crucial roles in this process^3^. The HBV genome is a 3.2 kB circular DNA, which is partially double-stranded, containing four overlapping genes: S/preS, C/preC, P, and X^4^. The X gene encodes a 17 kD protein called HBV X (HBx), which is a multifunctional regulator of transcriptional regulation, apoptosis, and cell cycle^5^. Among these functions, the transcriptional regulation may play an important role in HBV infection-induced hepatic injury, because HBx activates numerous signaling pathways linked to inflammation, immune response, and cell deaths^6,7^.

Protein phosphatase 2 A (PP2A) is a major serine/threonine phosphatase involved in regulating many cellular phosphorylation signals that are important for regulation of cell cycle, apoptosis, response to stress, and tumor suppression^8^. PP2A consists of holoenzyme complexes containing a scaffolding subunit A, a catalytic subunit C, and a variable regulatory subunit B^9^. PP2A, relying on its B subunit’s specificity, regulates multiple cellular signaling pathways^10^. PP2A-B56γ (B56γ), encoded by the *PPP2R5C* gene, is one of four isoforms (α, β, γ, and δ) of the PP2A regulatory B56 subunit^11,12^. It is reported that B56γ dephosphorylates p53 at Thr55 to stabilize p53 and promotes cell cycle arrest in human bone osteosarcoma epithelial U-2 OS cells^13^. Chen et al.^14^ demonstrated that B56γ of the PP2A holoenzyme was replaced by Simian virus 40 (SV40) small T antigen to facilitate cellular transformation.

Many viruses, from polyomaviruses to retroviruses, deregulate cellular signaling of host cells by using viral proteins to target PP2A, which is an abundant multifunctional cellular protein^15^. For instance, structural and biochemical studies revealed that SV40 inhibit PP2A activity via small T antigen’s N-terminal J domain^16^. HBx protein is also reported to directly interact with the PP2A-C subunit in HCC cells^17^. However, up to date, there is no report on the association between HBx and PP2A-B subunits. In the present study, we seek to investigate whether B56γ is targeted by HBx and to elucidate the regulatory roles in hepatic injury and mechanisms involved.

In the current study, we have demonstrated that B56γ was upregulated and positively correlated with HBx expression in the specimens of liver diseases’ patients, HBV-infected primary human hepatocytes (PHHs) in human-liver-chimeric (HLC) mice, HBx-transgenic (Tg) mice, HBV-infected HepG2 cells expressing sodium taurocholate cotransporting polypeptide (NTCP), and several HBx-expressing hepatic cells. Further, B56γ was increased to induce apoptosis of HBx-expressing hepatic cells through cell cycle arrest that is regulated by endoplasmic reticulum (ER) stress. Our study provided mechanistic insight into the pro-apoptotic function of B56γ in HBx-expressing hepatic cells and indicated that B56γ could be a potential therapeutic target for HBV-related hepatic injury.

## Results

### B56γ gene expression is upregulated in chronic hepatitis B patients

**Fig. 1:**
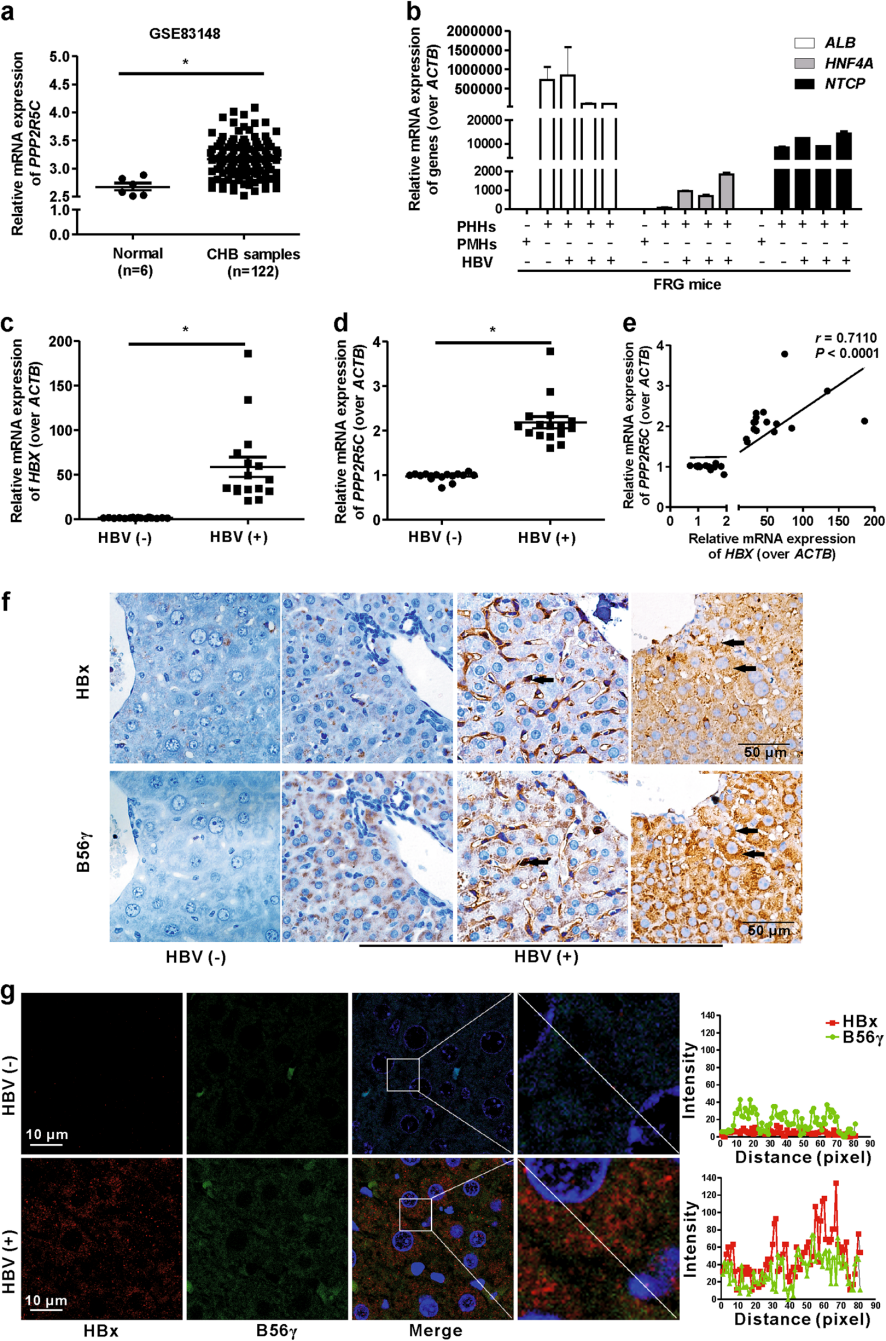
Expression of B56γ is elevated in liver tissues from chronic hepatitis B patients and HBV-infected primary human hepatocytes from HLC mice. **a** In a cohort from GEO database (Accession No. GSE83148), the mRNA level of *PPP2R5C* was higher in the liver tissues from chronic hepatitis B (CHB) patients (*n* = 122) than that in normal participants (*n* = 6). * *P* *<* 0.05 as compared with normal control. **b** The mRNA levels of human *ALB*, *HNF4A*, and *NTCP*, as human hepatocyte markers, were elevated in primary human hepatocytes (PHHs) from livers of HLC mice with either HBV infection (HBV(+)) or uninfection (HBV(−)) compared with primary mouse hepatocytes (PMHs) using qPCR. *ACTB* was used as the control, *n* = 3. The mRNA levels of *HBX* **c** and *PPP2R5C* **d** were elevated in HBV-infected PHHs from HLC mice measured by qPCR. *ACTB* was used as the control, *n* = 16. **e** Pearson’s correlation analysis for *HBX* and *PPP2R5*C mRNA in HBV-infected/uninfected PHHs from HLC mice. **f** IHC images show that the levels of HBx and B56γ proteins are increased in HBV-infected PHHs from HLC mice. Arrows indicate colocalization of HBx and B56γ. Scale bar = 50 μm. **g** The fluorescence for HBx and B56γ colocalization in HBV-infected/uninfected livers of HLC mice. Left, the fluorescence images; Scale bar = 10 μm. Right, fluorescence intensity curves of HBx (red) and B56γ (green) proteins in hepatocytes. Fluorescence curves were analyzed with line intensity profile by Zen 2012 software

In order to explore the relationship between *PPP2R5C* (encoding B56γ) expression and HBV infection, a genomic expression data set of chronic hepatitis B (CHB) patients was employed. In one cohort from Gene Expression Omnibus (GEO) database (Accession No. GSE83148; https://www.ncbi.nlm.nih.gov/geo/), the mRNA expression of *PPP2R5C* was significantly higher in the liver tissues of CHB patients than that in normal participants (Fig. 1a).

### B56γ positively correlates with HBx in animal models

As B56γ expression had been proven to increase in CHB patients, we further studied the relationship of HBx with B56γ in HBV-infected PHHs from HLC mice and HBx-overexpressing hepatocytes from HBx-Tg mice. First, we constructed the HLC mice for HBV infection through transplantation of PHHs into Fah^−/−^/Rag2^−/−^/Il2rg^−/−^ (FRG) mice. Human hepatocytes were then transplanted into FRG mice. FRG mice with high human hepatocyte-repopulation were employed to be infected with HBV for 6 weeks. Thereafter, we measured the gene expression of human hepatocyte markers (*ALB*, *HNF4A*, and *NTCP*), and *HBX* and *PPP2R5C* in the liver of HLC mice. The significant upregulation of the mRNA levels of *ALB*, *HNF4A*, and *NTCP* were observed in PHHs from HLC (FRG with PHHs transplantation) mice compared with primary mouse hepatocytes from FRG mice (Fig. 1b). As shown in Fig. 1c, d, the gene expression levels of *HBX* and *PPP2R5C* were higher in the livers of HLC mice with HBV infection than those in mice without infection. Moreover, there was a significant correlation (*r* *=* 0.7110, *P* *<* 0.0001) between *HBX* and *PPP2R5C* in PHHs from HLC mice (Fig. 1e). Immunohistochemistry (IHC) (Fig. 1f) analysis showed a colocalized expression of B56γ and HBx in the hepatocytes of HLC mice with HBV infection. Furthermore, fluorescence curves analyzed from immunohistofluorescence images with line intensity profile by Zen 2012 software also presented similar expression pattern of B56γ and HBx in the hepatocytes of HLC mice with HBV infection (Fig. 1g). Meanwhile, the Pearson’s correlation for the colocalization of B56γ and HBx proteins reach 0.7966. In contrary, there was a little background noise signal of HBx and significant lower signal of B56γ in HBV-uninfected HLC mice. These results indicated the colocalization of B56γ and HBx in HBV-infected hepatic cells.

**Fig. 2:**
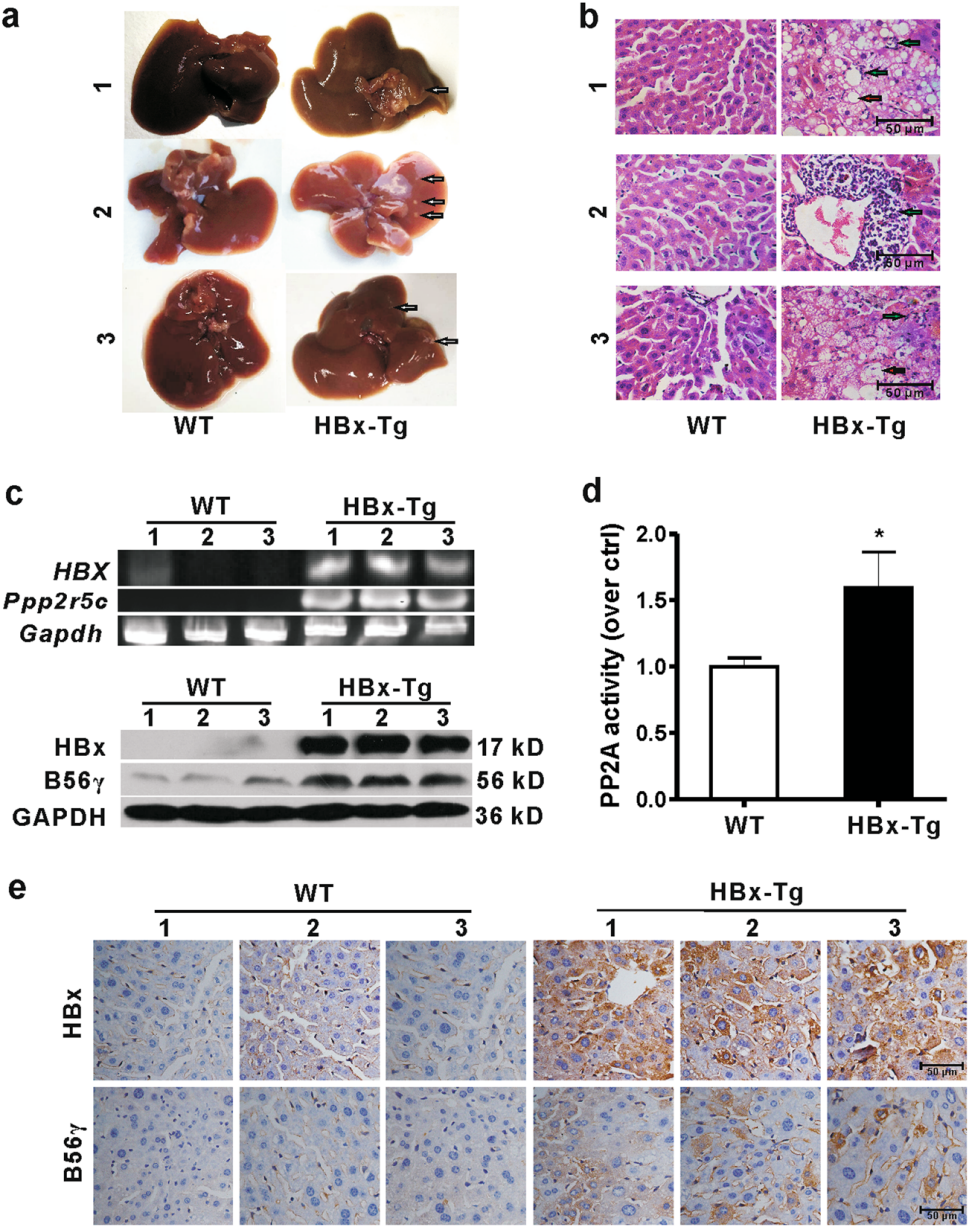
Expression of B56γ is upregulated in livers from HBx-Tg mice. The livers were excised from wild type (WT) and HBx-Tg mice (12 months old). *n* = 3. **a** Gross observation of livers show that HBx-Tg mice spontaneously develop hepatic hyperplastic nodules (white arrows). **b** Hepatic inflammation and steatosis are observed in HE-stained liver sections from HBx-Tg mice. Green arrows indicate the infiltration of neutrophils. Red arrows indicate hepatic lipid vacuoles. **c** The *HBX* mRNA transcript and the mRNA level of *Ppp2r5c* increased in the livers of HBx-Tg mice analyzed using RT-PCR. *Gapdh* was used as the internal control (upper panel). The HBx protein expressed in the livers of HBx-Tg mice. The level of B56γ protein elevated in the livers of HBx-Tg mice compared with those in WT mice as examined by Western blot (lower panel). GAPDH was used as the loading control. **d** The protein phosphatase activity of PP2A was determined with a Serine/Threonine Phosphatase Assay Kit. *P *<* 0.05 as compared with WT mice. **e** The distribution and levels of HBx and B56γ proteins were higher in hepatocytes of HBx-Tg mice comparing with those in WT mice as examined by IHC. Brown staining indicates immunoreactivity. Scale bar = 50 μm

A previous study showed that hepatic injury and hyperplastic nodules were observed in 12 months old HBx-Tg mice^18^. Thus, in the present study, we chose 12 months old HBx-Tg mice to explore the role of B56γ in HBV-related hepatic injury. As we expected, several hepatic hyperplastic nodules were observed in the liver from HBx-Tg mice (Fig. 2a). Hepatic infiltration of neutrophils and lipid vacuoles were observed in liver sections from HBx-Tg mice using hematoxylin and eosin (HE) staining (Fig. 2b). As shown in Fig. 2c, the levels of *Ppp2r5c* mRNA (upper panel) and B56γ protein (lower panel) were increased in the livers of HBx-Tg mice compared with those of wild type (WT) mice. Moreover, the protein phosphatase activity of PP2A was upregulated in HBx-Tg mice (Fig. 2d). IHC staining showed higher expression of B56γ and HBx in the livers of HBx-Tg mice (Fig. 2e). These data support a strong correlation between B56γ and HBx in HBV-infected or HBx-overexpressing livers from HBV-infected HLC or HBx-Tg mice, respectively.

### B56γ is upregulated by HBx in vitro

**Fig. 3:**
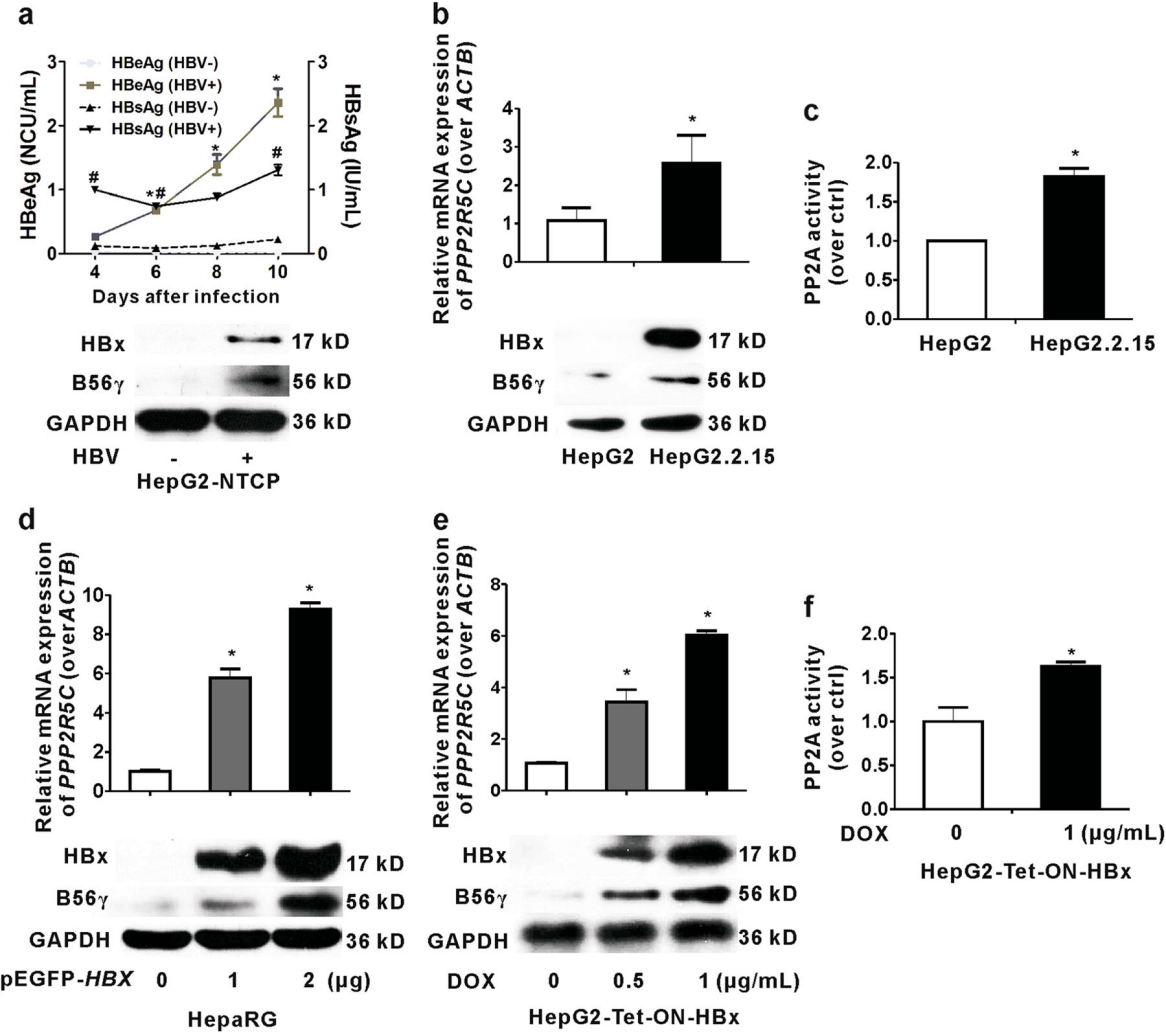
B56γ is upregulated in various HBx-expressing hepatic cell lines. **a** Levels of HBeAg and HBsAg secretion in HepG2-NTCP cells infected with HBV for 10 days were detected using chemiluminescence (upper panel, * *P* *<* 0.05 as compared with HBeAg in the HBV-uninfected group ^#^; *P* *<* 0.05 as compared with HBsAg in the HBV-uninfected group). The level of B56γ protein was upregulated in HBV-infected HepG2-NTCP cells with HBx expression (lower panel). **b** The levels of *PPP2R5C* mRNA and B56γ protein were upregulated in HepG2.2.15 cells with HBx expression, compared with HepG2 cells as control (*P *<* 0.05) using qPCR (upper panel) and western blot (lower panel). **c** The PP2A activity was higher in HepG2.2.15 cells than in HepG2 cells with Serine/Threonine Phosphatase Assay Kit (*P < 0.05). **d** The levels of *PPP2R5C* mRNA and B56γ protein were increased in differentiated HepaRG cells transfected by pEGFP-*HBX* plasmids with higher HBx expression. The values were measured using qPCR (upper panel) and western blot (lower panel). *P *<* 0.05 as compared with the pEGFP vector group. **e** The levels of *PPP2R5C* mRNA and B56γ protein were elevated in DOX-treated HepG2-Tet-ON-HBx cells with higher HBx expression. *P *<* 0.05 as compared with DOX-untreated cells. *ACTB* was used as qPCR reference. GAPDH protein was used as protein loading control. **f** The protein phosphatase activity of PP2A was upregulated by DOX (1 μg/mL) treatment of HepG2-Tet-ON-HBx cells. *P *<* 0.05 as compared with the DOX-untreated cells

To assess whether B56γ expression positively correlates with HBx in vitro, we measured the B56γ expression in HBV-infected HepG2 cells, which overexpress NTCP (named as HepG2-NTCP) and HBV genome-integrated HepG2.2.15 cells. NTCP, the natural binding receptor of HBV in human hepatocytes^19^, expresses to facilitate HBV infection in HepG2-NTCP cells. As shown in Fig. 3a, hepatitis B surface antigen (HBsAg) and hepatitis B e antigen (HBeAg) were upregulated in the supernatants of HepG2-NTCP cells after HBV infection from the 4th day to the 10th day. We collected the cell lysis on the 10th day and found that HBx and B56γ proteins were also increased by HBV infection in HepG2-NTCP cells. HepG2.2.15 cells, constructed with two head-to-tail copies of the HBV genome inserted into HepG2 cells, support stable HBV replication and virus particle production^20^. Because of its persistent expression of HBV proteins, HepG2.2.15 is an ideal cell model for exploring the molecular mechanism associated with HBV and its proteins^21^. As shown in Fig. 3b, the transcription and translation levels of B56γ were elevated in HepG2.2.15 cells compared with the control HepG2 cells. We further detected the upregulation of PP2A activity in HepG2.2.15 cells through serine/threonine phosphatase assay (Fig. 3c). From the results of HepG2-NTCP and HepG2.2.15 cells, we observed that HBV infection induced B56γ expression in hepatic cells.

Then, we explored whether HBx contributes to the upregulation of B56γ in two HBx-expressing hepatic cells, including HepaRG and HepG2-Tet-ON-HBx cells^22^. The mRNA and protein levels of B56γ were significantly elevated with HBx expression in the differentiated hepatocyte-like HepaRG cells transfected with pEGFP-*HBX* plasmids (Fig. 3d). In HepG2-Tet-ON-HBx cells, the expression of HBx can be switched on by doxycycline (DOX) treatment. The mRNA and protein levels of HBx and B56γ were increased in HepG2-Tet-ON-HBx cells in a DOX concentration-dependent manner (Fig. 3e). In addition, PP2A activity was significantly increased in HepG2-Tet-ON-HBx cells upon DOX (1 μg/mL) induction (Fig. 3f). These results lead us to conclude that HBx induces the transcription, translation of B56γ, and increases PP2A activity in hepatic cells with HBV infection or HBx expression.

### HBx upregulates B56γ through AP-1 transactivation that is dependent on ER stress

**Fig. 4:**
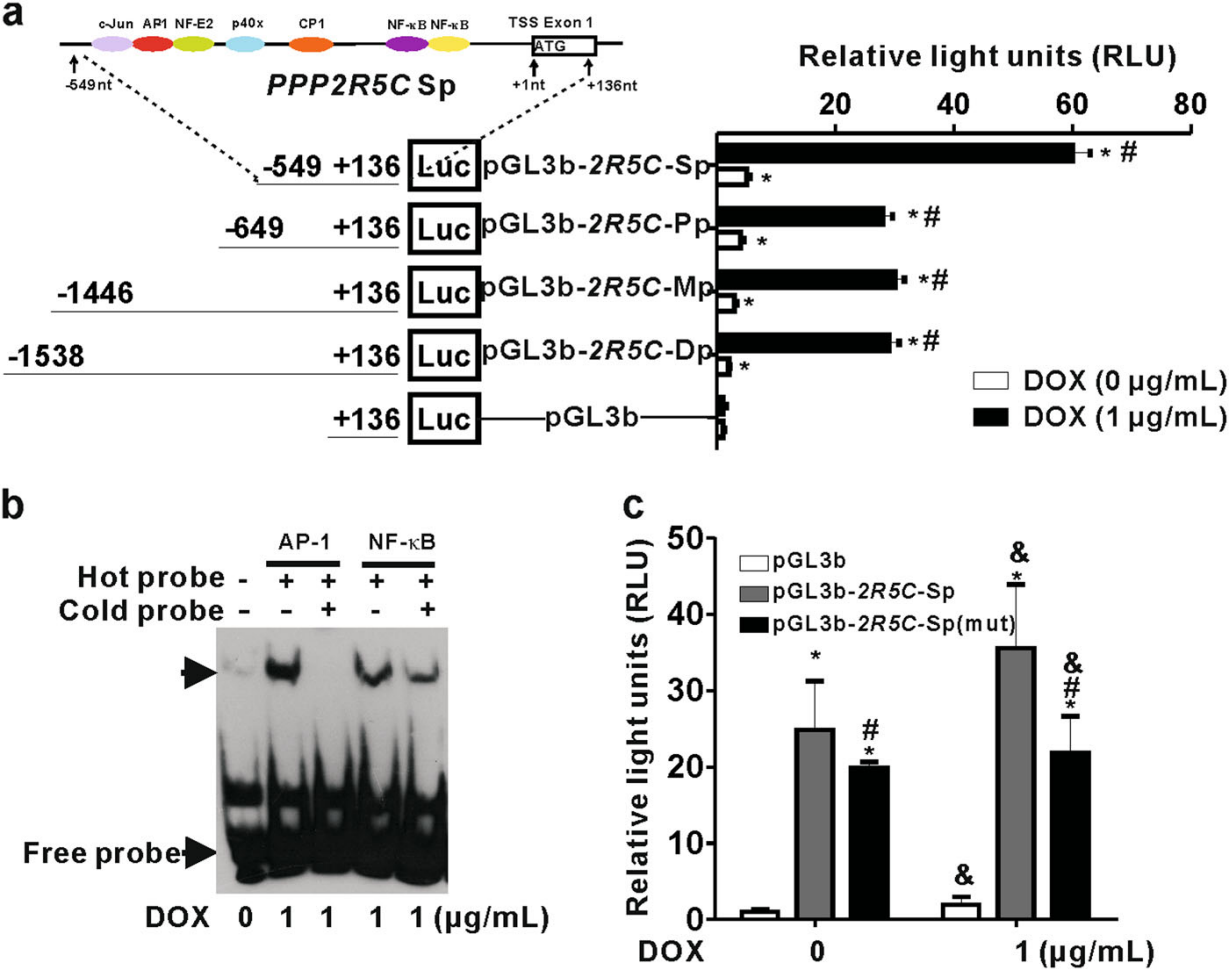
B56γ is upregulated via AP-1 transactivation in HBx-expressing cells. **a** Transcriptional activities of the different length’s fragments from the 5′-flanking region of *PPP2R5C* promoter were determined by the luciferase reporter genes in HepG2-Tet-ON-HBx cells. The upper panel is a scheme for the transcription factor-binding sites located at 5′-flanking region of *PPP2R5C* short promoter (Sp). The fragments of the *PPP2R5C* 5′-flanking region include various lengths of promoter, including nucleotides −1701 ~ +136 nt (Dp), −1446 ~ +136 nt (Mp), −694 ~ +136 nt (Pp), and −549 ~ +136 nt (Sp) were constructed into pGL3-basic plasmids (pGL3b). Transcription start site (TSS) is named as +1 nt. Each construct or pGL3b vector with pRL-TK plasmids were transfected into HepG2-Tet-ON-HBx cells. The luciferase activities were measured using Dual-Luciferase Reporter Assay Kit. The relative luciferase activity expressed as relative light units (RLU) was normalized to *Renilla* activity and was relative to the activity of pGL3b, which was set as 1. pGL3b-*2R5C*-Sp had the strongest transcriptional activity. *P *<* 0.05 as compared with pGL3b control.^#^ *P* *<* 0.05 as compared with no DOX treatment in the same constructs. **b** EMSA of *PPP2R5C* core short promoter region. 5′-biotin-labeled double-stranded oligonucleotides probes (Hot probes; lanes 2–3, for AP-1; lane 4–5, for NF-κB) were incubated with 10 μg of nuclear protein. Arrow (upper) indicates the DNA-protein complex. Arrow (lower) indicates the free probe. For competition assay, the specific competitors contained 200-time molar excess of unlabeled probes (Cold probes; lane 3, for AP-1; lane 5, for NF-κB). The EMSA image shows that AP-1, but not NF-κB, binds to the core short promoter region of *PPP2R5C* in DOX-induced HepG2-Tet-ON-HBx cells. **c** Transcriptional activities of the *PPP2R5C* core short promoter (pGL3b-*2R5C*-Sp) and short promoter with AP-1 binding site mutation (pGL3b-*2R5C*-Sp(mut)) were measured by the luciferase reporter genes in HepG2-Tet-ON-HBx cells. The relative luciferase activity of pGL3b-*2R5C*-Sp increased by HBx was partially decreased by introducing mutation at the AP-1 binding site. *P *<* 0.05 as compared with pGL3b control. ^#^*P* *<* 0.05 as pGL3b-*2R5C*-Sp(mut) compared with pGL3b-*2R5C*-Sp. ^&^*P* *<* 0.05 as compared with cells untreated with DOX

Reports have shown that HBx-regulated gene expression is mostly mediated by transcription factors^23,24^. HBx-associated transcription process of *PPP2R5C* was speculated via transcriptional regulation, so we identified the transcription factor-binding sites at the *PPP2R5C* promoter regions. The transcription factors that bind to different 5′-flanking regions of *PPP2R5C* promoter were listed in Table S1 and depicted in Fig. 4a, including nucleotides −1701 ~ +136 nt (distant promoter, Dp), −1446 ~ + 136 nt (middle promoter, Mp), −694 ~ +136 nt (proximal promoter, Pp), and −549 ~ +136 nt (short promoter, Sp). The control pGL3-basic (pGL3b) vector, and the constructed recombinant plasmids pGL3b-*2R5C*-Dp, -Mp, -Pp, or -Sp containing transcription factor-binding sites were transiently transfected into HepG2-Tet-ON-HBx cells. Then, the transcriptional activities of these recombinant plasmids were determined using luciferase reporter assay. As shown in Fig. 4a, pGL3b-*2R5C*-Sp had the maximum transcriptional activity, suggesting that *PPP2R5C* transcriptional activity was mainly controlled by the central promoter at the short 5′-flanking region (−549 ~ +136 nt).

Next, we searched the transcription factors that potentially bind to the *PPP2R5C*-Sp region using an online gene-regulation prediction tool. Various transcription factors including nuclear factor-kappa B (NF-κB) and activator protein-1 (AP-1) were identified to bind the Sp region. We performed the electrophoretic mobility shift assay (EMSA) to test whether NF-κB or AP-1 could bind to the Sp region of *PPP2R5C*. As shown in Fig. 4b, in DOX-treated HepG2-Tet-ON-HBx cells, the biotin-labeled probe (Hot probe) of AP-1 binding to the Sp region competed with AP-1-unlabeled probe (Cold probe), but this was not the case for NF-κB. To determine the role of AP-1, we conducted the transcriptional activity assay with pGL3b-*2R5C-*Sp and pGL3b-*2R5C*-Sp(mut) (mutant at AP-1-binding site). It was found that this mutation significantly decreased the transcriptional activity of *PPP2R5C*-Sp region (Fig. 4c), demonstrating the importance of AP-1 binding on the *PPP2R5C* promoter for its transactivation. AP-1 is a heterodimer formed by c-Fos and c-Jun^25^. Further, we found that HBx increased c-Jun expression and induced its translocation into the nucleus in HepG2-Tet-ON-HBx cells upon DOX induction using immunofluoresence assay (Figure S1). These results suggest that the upregulation of B56γ triggered by HBx may via *PPP2R5C* transactivation with AP-1 being the transcription factor.

**Fig. 5:**
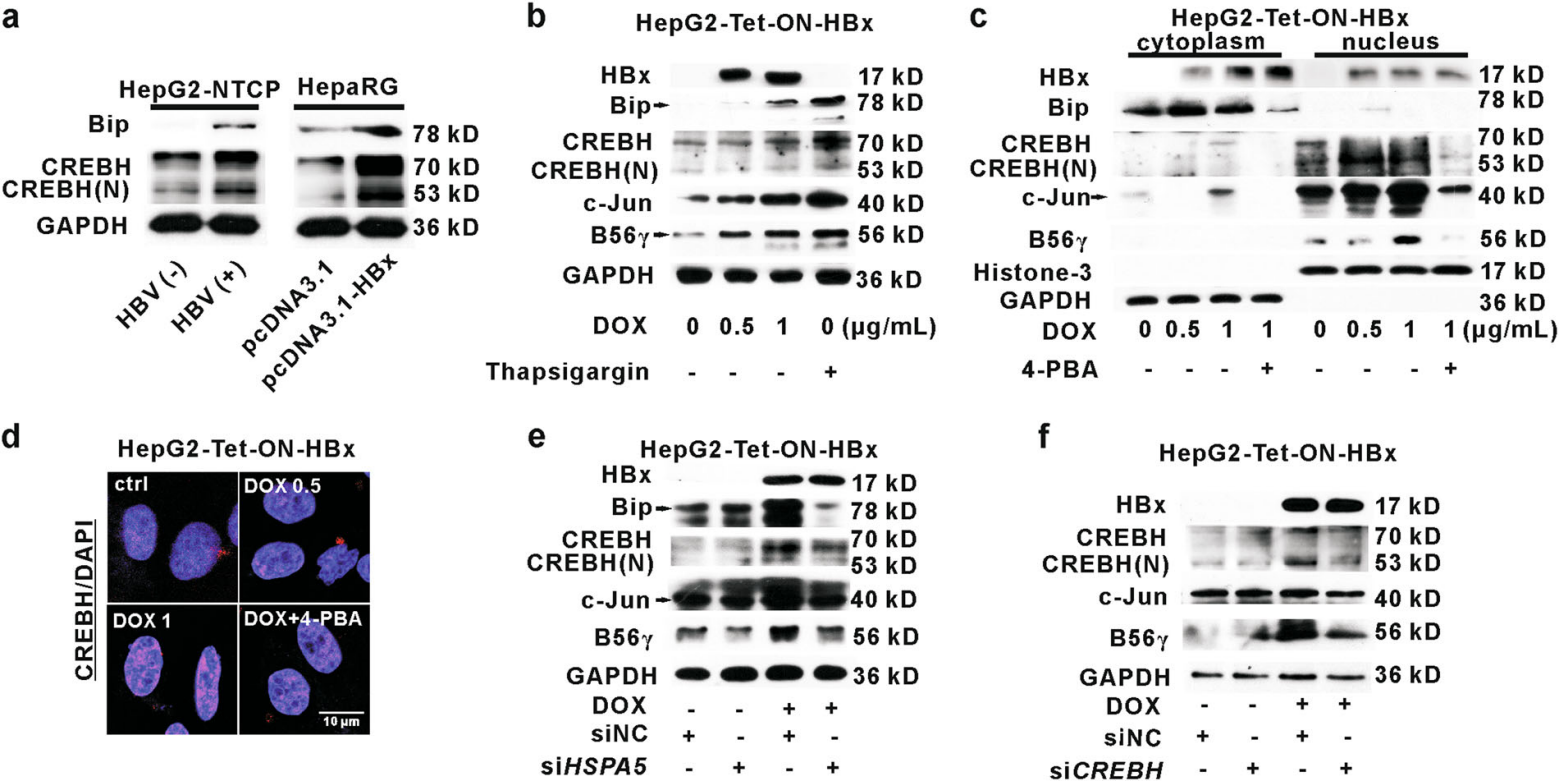
ER stress mediates B56γ upregulation via AP-1 transactivation in HBx-expressing hepatic cells. **a** ER stress in HBV-infected HepG2-NTCP cells and HBx-expressing HepaRG cells. The ER stress-related proteins, Bip and CREBH(N), were upregulated in HBV-infected HepG2-NTCP cells and HepaRG cells with pcDNA3.1-*HBX* plasmids transfection. **b** C-Jun and ER stress-related proteins, Bip and CREBH(N), were upregulated in DOX-induced HepG2-Tet-ON-HBx cells with higher HBx expression. Thapsigargin is a positive control for ER stress induction. **c** The cytoplasmic and nuclear fraction of HepG2-Tet-ON-HBx cells upon DOX induction were extracted for analyzing CREBH(N) and c-Jun activation. 4-PBA, an ER stress inhibitor, decreased the level of CREBH(N) and blocked it translocation into nucleus as detected by western blot. **d** Representative confocal images presented the CREBH(N) translocation into nucleus in HepG2-Tet-ON-HBx cells upon 1 μg/mL DOX treatment, whereas 4-PBA blocked its translocation. Scale bar = 10 μm. **e**, **f** Upon 1 μg/mL DOX induction, siRNA for *HSPA5* (**e**) or *CREBH* (**f**) was transfected into HepG2-Tet-ON-HBx cells for 8 h. After another 24 h, AP-1, Bip, CREBH(N), and B56γ were elevated by DOX, which were rescued by si*HSPA5* or si*CREBH*, as detected by Western blot

Previous studies have shown that HBV or HBx induced ER stress in some experimental models^26,27^. CREBH (cAMP-responsive element binding protein, hepatocyte specific) is a liver homolog of ATF6 (activating transcription factor 6), one of the three major ER stress branches^27^. We found that the levels of binding immunoglobulin protein (Bip, an indicator of ER stress) and CREBH activated form, CREBH nucleus form (CREBH(N)), were upregulated in HBV-infected HepG2-NTCP cells and HBx-expressing HepaRG cells (Fig. 5a). This result suggested that HBx could induce ER stress CREBH signaling in HBV-infected or HBx-expressing hepatic cells. In order to further confirm that HBx contributed to ER stress occurrence, we detected ER stress in HepG2-Tet-ON-HBx cells. We found that HBx expression also induced upregulation of Bip, CREBH(N), B56γ, and c-Jun (a component of AP-1) (Fig. 5b). Thapsigargin, a typical ER stress inducer, was used as a positive control. On the other hand, to explore whether AP-1 was regulated by ER stress, we studied AP-1 activation by blocking ER stress with its inhibitor, 4-phenylbutyric acid (4-PBA), in HepG2-Tet-ON-HBx cells. As shown in Fig. 5c, the upregulation of c-Jun and B56γ caused by HBx was attenuated by 4-PBA. Immunofluorescence analysis confirmed that HBx-triggered CREBH activation was rescued by 4-PBA (Fig. 5d). As a major ER chaperone, Bip regulates the activation of ER stress^28^. Knockdown of Bip with si*HSPA5* attenuated the activation of CREBH and the increase of c-Jun and B56γ in DOX-treated HepG2-Tet-ON-HBx cells (Fig. 5e). Moreover, knockdown of CREBH with si*CREBH* relieved HBx-induced ER stress, c-Jun activation, and B56γ elevation (Fig. 5f). In summary, HBx-triggered *PPP2R5C* expression is thought to be regulated by AP-1 transactivation and this process is mediated by ER stress CREBH signaling.

### B56γ mediates HBx-induced hepatic cell cycle arrest through p53-dependent pathway

**Fig. 6:**
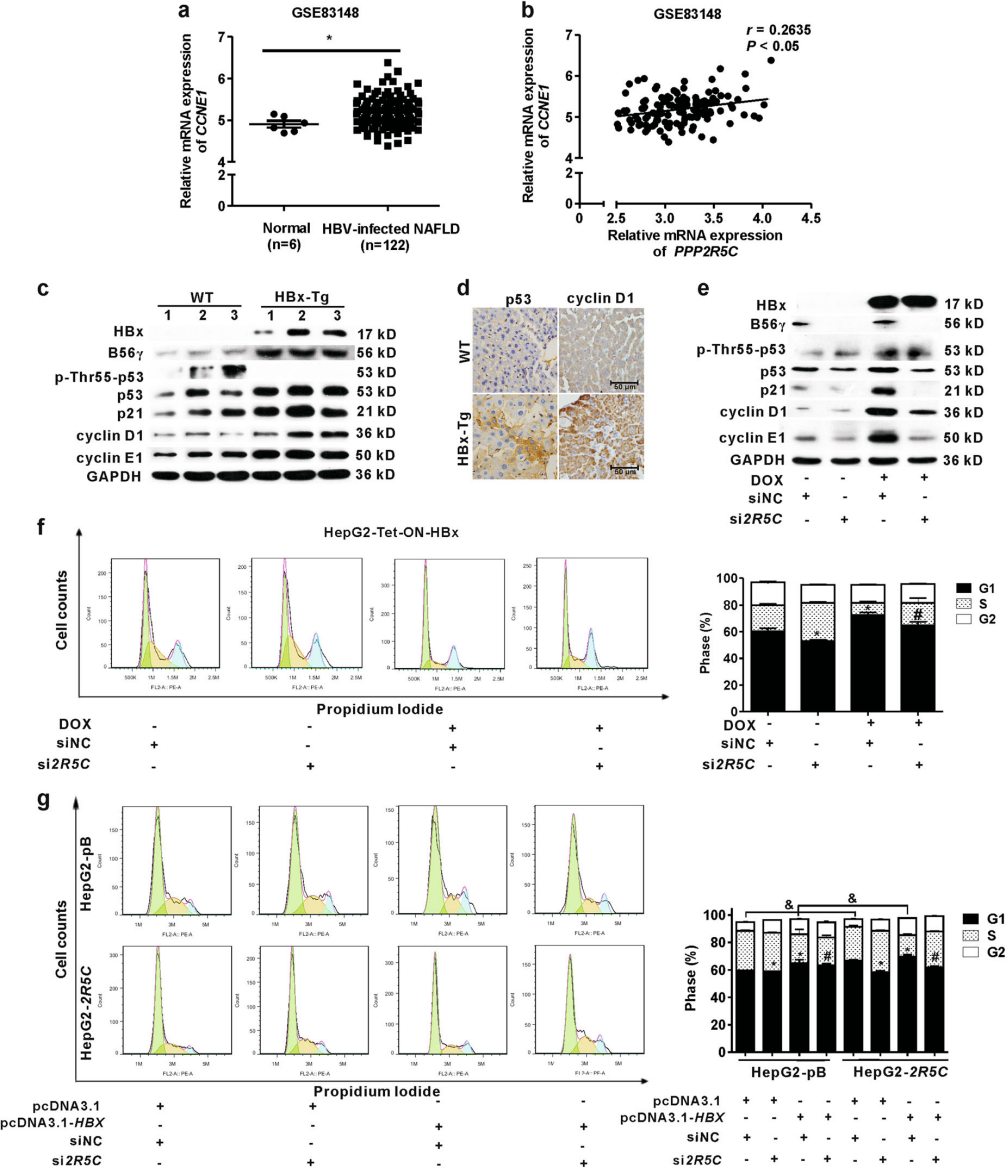
B56γ regulates p53-related cell cycle in HBx-Tg mice and HBx-expressing cells. **a** In a cohort from GEO database (Accession No. GSE83148), the mRNA level of *CCNE1* was higher in the liver tissues from CHB patients (*n* = 122) than that in normal participants (*n* = 6). * *P* *<* 0.05 as compared with normal control. **b** Pearson’s correlation analysis was used for the relationship of *PPP2R5C* and *CCNE1* mRNA expression. **c** Expressions of HBx, B56γ, p-Thr55-p53, p53, p21, cyclin D1, and cyclin E1 proteins in the livers of wild type (WT) and HBx-Tg mice were detected by western blot. **d** Representative IHC images show the levels and distributions of p53 and cyclin D1 in the livers of WT and HBx-Tg mice. Scale bar = 50 μm. **e**, **f** HepG2-Tet-ON-HBx cells upon DOX (1 μg/mL) treatment were transfected with *PPP2R5C* siRNA for 8 h. **e** After another 24 h, the expressions of HBx, B56γ, p-Thr55-p53, p53, p21, cyclin D1, and cyclin E1 proteins were detected by western blot. **f** Cell cycle distribution analysis for HepG2-Tet-ON-HBx cells with *PPP2R5C* siRNA transfection was detected using FCM. Cell cycle distribution images (left panel) and bar graphs (right panel) were presented. *P *<* 0.05 as compared with siNC. ^#^*P* *<* 0.05 as compared with siNC with DOX induction. **g** HepG2-pB and HepG2-*2R5C* cells were transfected with pcDNA3.1-*HBX* or pcDNA3.1 plasmids combined with si*2R5C* or siNC for 8 h. Another 24 h later, the cells were analyzed by FCM. Cell cycle distribution images (left panel) and bar graphs (right panel) were presented. *P *<* 0.05 as compared with siNC treatement with pcDNA3.1 transfection. ^#^*P* *<* 0.05 as compared with siNC upon pcDNA3.1-*HBX* transfection. ^&^*P* *<* 0.05 as HepG2-*2R5C* compared with HepG2-pB cells

As we have understood that B56γ positively correlates with HBx expression in HBV-infected hepatocytes from HLC mice, HBx-Tg mice, and HBx-expressing hepatic cells, the role and mechanism of B56γ in HBV-related hepatic injury were further investigated. Here, in one cohort from GEO database (Accession No. GSE83148), the mRNA level of *CCNE1* (encoding cyclin E1, a protein which regulates cell cycle) was higher in CHB patients than that in normal participants (Fig. 6a). What is more, *CCNE1* expression was positively correlated with the mRNA level of *PPP2R5C* (*r* *=* 0.2635, *P* *<* 0.05) (Fig. 6b). Further, the levels of p21, cyclin D1, and cyclin E1 proteins were higher in HBx-Tg mice than those in WT mice (Fig. 6c). It has been shown that PP2A harboring B56γ subunit can activate p53 by dephosphorylating p53 at Thr55 site^13^. In HBx-Tg mice, the level of p-Thr55-p53 was decreased, whereas the expressions of total p53 and B56γ proteins were increased (Fig. 6c). IHC analysis also showed that the signals of p53 and cyclin D1 proteins were stronger in the liver of HBx-Tg mice than those in WT mice (Fig. 6d). These data indicate that HBx disturbs cell cycle progression involved in p53 signaling through PP2A-B56γ in hepatocytes from clinical samples and animal models.

In vitro, p53, p21, cyclin D1, and cyclin E1 proteins were positively correlated, whereas p-Thr55-p53 was negatively correlated, with HBx and B56γ proteins in DOX-treated HepG2-Tet-ON-HBx cells (Fig. 6e). *PPP2R5C* small interfering RNA (si*2R5C*) decreased B56γ protein level, but it had no effect on-HBx expression in HepG2-Tet-ON-HBx cells. The levels of p53, p21, cyclin D1, and cyclin E1 proteins were downregulated and the p-Thr55-p53 level was upregulated by si*2R5C* (Fig. 6e). Cell cycle pattern was analyzed using flow cytometry (FCM). As shown in Fig. 6f, HBx led to G1 phase ratio increase in HepG2-Tet-ON-HBx cells upon DOX induction. In contrast, si*2R5C* alleviated the G1 phase arrest caused by HBx (Fig. 6f). In addition, as shown in Fig. 6g, HepG2-*2R5C* cells presented higher G1 phase ratio than the control cells. Moreover, si*2R5C* relieved the G1 phase ratio increase in pcDNA3.1-*HBX-*transfected HepG2-*2R5C* cells. We also measured the cell viability of HepG2-Tet-ON-HBx cells with MTS assay. As shown in Figure S2a, the cell viability was decreased by DOX induction in a concentration-dependent manner. Moreover, the decrease in cell viability caused by HBx expression was partially recovered by knockdown of B56γ with si*2R5C* (Figure S2b). Taken together, our results demonstrate that HBx-triggered B56γ elevation regulates p53-mediated cell cycle G1 phase arrest and cell viability decrease in HBV-infected or HBx-expressing hepatocytes.

### B56γ, induced by HBx, regulates apoptosis both in vivo and in vitro

**Fig. 7:**
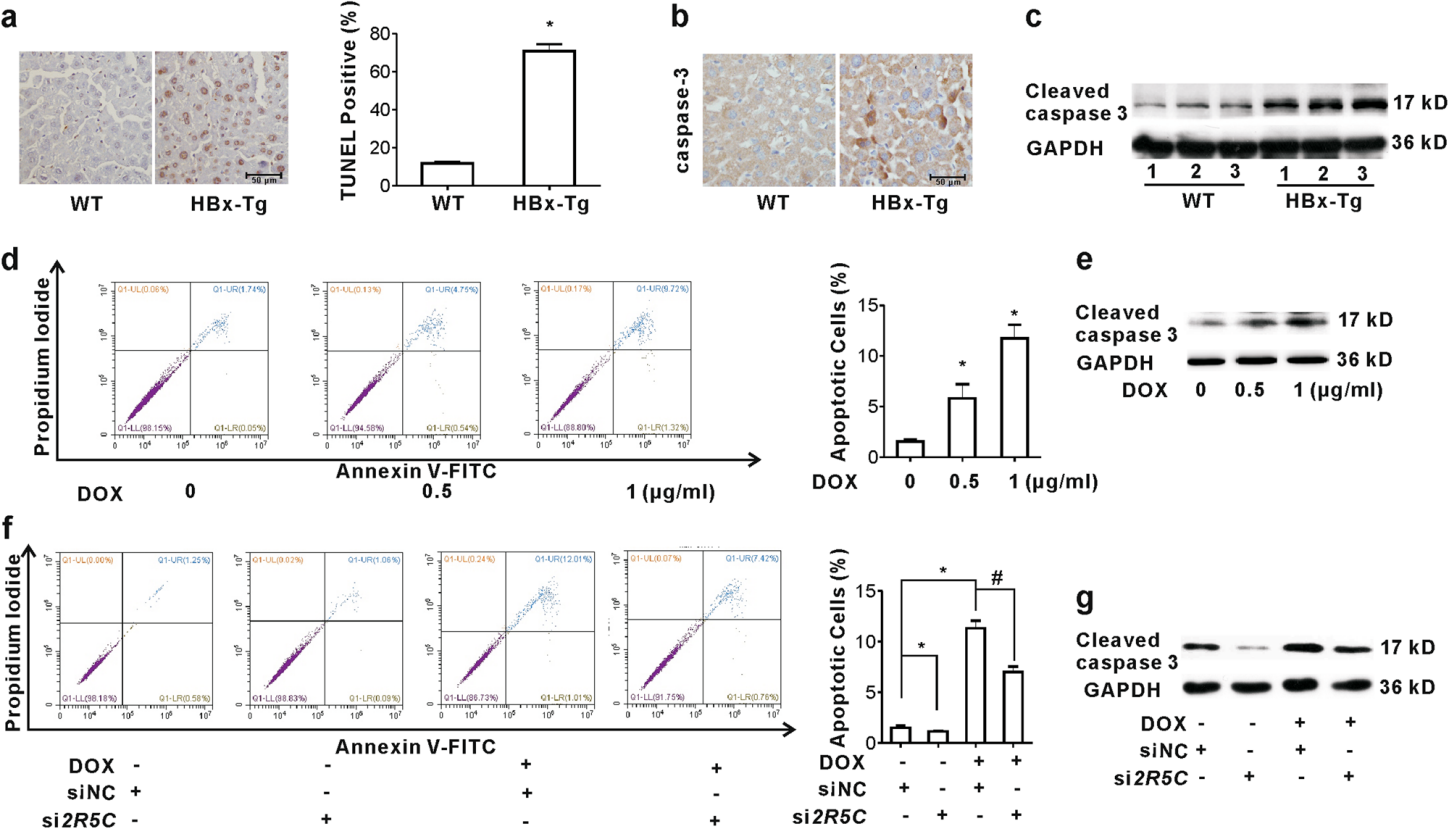
HBx triggers apopotosis through upregulation of B56γ in hepatocytes. **a**–**c** In the HBx-Tg mice, **a** TUNEL assay for apoptosis (left panel, IHC images; right panel, bar graphs). *P *<* 0.05 as compared with wild type (WT) mice. **b** the level of caspase 3 was detected using IHC. Representative images were shown, scale bar = 50 μm. (**c**) Cleaved caspase 3 protein was detected using western blot. **d**, **e** In HepG2-Tet-ON-HBx cells upon DOX (0.5 and 1 μg/mL) induction, **d** the apoptotic cells were measured with Annexin-V-PI Kit using FCM (left panel, FCM images; right panel, bar graphs). *P *<* 0.05 as compared with control. **e** Cleaved caspase 3 protein was detected using western blot. **f**, **g** Before induced by DOX (1 μg/mL), HepG2-Tet-ON-HBx cells were transfected with si*2R5C* or siNC. **f** The apoptotic cells were measured with Annexin-V-PI Kit using FCM (left panel, FCM images; right panel, bar graphs), *P *<* 0.05 as compared with siNC. ^#^*P* *<* 0.05 as compared with siNC with DOX induction. **g** Western blot for cleaved caspase 3 protein

According to the evidence that cell cycle arrest associates with apoptosis^29^, we investigated whether HBx-induced apoptosis was related with the regulation of B56γ. Compared with WT mice, HBx-Tg mice presented more TdT-mediated dUTP nick-end labeling (TUNEL) staining positive cells (Fig. 7a), higher caspase 3 protein staining signals (Fig. 7b) and expression level (Fig. 7c). In HepG2-Tet-ON-HBx cells, the ratio of late apoptotic cells (Annexin V^+^/PI^+^) was significantly upregulated by HBx expression in dose-dependent pattern (Fig. 7d). Consistently, the level of cleaved caspase 3 was also increased (Fig. 7e). In order to explore whether the pro-apoptotic function of HBx was regulated by B56γ, knockdown assay was preformed. As Fig. 7f and g shown, B56γ knockdown with si*2R5C* attenuated the elevation of ratio of late apoptotic cells and the level of cleaved caspase 3 triggered by HBx induction. Moreover, we conducted experiments to determine the contribution of ER stress to HBx-induced apoptosis. We measured apoptosis after blocking ER stress with 4-PBA or si*CREBH* in HBx-expressing hepatic cells. As shown in Figures S3a and S3c, FCM results demonstrate that both 4-PBA and si*CREBH* significantly decreased the ratio of late apoptotic cells induced by HBx expression. Moreover, the expression of cleaved caspase 3 was also examined in DOX-induced HepG2-Tet-ON-HBx cells. These results show that both 4-PBA and si*CREBH* reduced the level of cleaved caspase 3 induced by HBx expression (Figures S3b and 3d). These data indicate that B56γ, induced by HBx, regulates apoptotic cell death through CREBH signaling both in HBx-Tg mice and HBx-expressing cells.

## Discussion

A common biological strategy of many viruses is to target PP2A and disturb signaling pathways in the host cells, based on interactions between viral proteins and PP2A holoenzyme subunits^15^. For example, the SV40 small T antigen binds directly to the PP2A subunit A and downregulates PP2A activity by displacing the B56γ subunit from the PP2A core enzyme, leading to cell transformation and tumorigenesis^14,16^. Human papillomaviruses E7 protein has been shown to bind PP2A-A/C core enzyme to activate the PI3K/AKT transforming pathway^30^. Human immunodeficiency virus transactivator Tat, a small regulatory protein, interacts with the promoters of *PPP2R5E* (coding subunit B’ε) and *PPP2R1B* (coding subunit Aβ) and promotes their transcription, leading to PP2A overexpression^31^. In this study, B56γ expression was proved to be positively correlated with HBx in HBV-infected PHHs from HLC mice as well as HBx-Tg mice and HBx-expressing cells. Also, we for the first time observed that HBx protein promotes *PPP2R5C* transcription, and the process is mediated by CREBH/AP-1 pathway, leading to upregulation of B56γ and PP2A holoenzyme activity to dephosphorylate p-Thr55-p53 involved in the HBV/HBx-mediated cell cycle arrest and apoptosis in hepatocytes.

With ubiquitous cellular expression, PP2A exerts roles in the control of cell proliferation, cell cycle arrest, cell migration, and survival. For instance, p53 is activated when it is dephosphorylated at Thr55 site by B56γ, leading to higher stability^13^, and activated p53 positively regulates cell cycle arrest through downstream protein p21^32^. Recently, p21 protein-activated kinase 1 (PAK1), involved in cell cycle arrest^33^, has been proven to be downregulated by *PPP2R5C* siRNA in Jurkat T cells^34^. It has been reported that HBx activates p53 to upregulate cyclin D1 in HBx-Tg mice^35^. In this study, the expression of p53, p21, cyclin D1, and cyclin E1 proteins positively correlated with B56γ protein expression in HBV-infected PHHs from HLC mice, and livers of HBx-Tg mice, respectively. Moreover, B56γ knockdown rescued the upregulation of p53, p21, cyclin D1, and cyclin E1 caused by HBx to relieve cell cycle arrest in hepatic cells. These results indicate that the pro-apoptotic function of B56γ may due to p53-dependent cell cycle regulation and G1 phase arrest through dephosphorylating p-Thr55-p53.

Previous studies showed that HBx regulates gene expression by transactivating some transcription factors, such as NF-κB and AP-1^36,37^, rather than by directly binding to DNA. In agreement with those findings, we observed that HBx significantly activated AP-1 and induced its translocation into the nuclei of hepatic cells. Also, we proved that HBx-activated AP-1 binds to the core region at the short 5′-flanking (−549 ~ +136 nt) of *PPP2R5C* gene promoter, initiating its transcription. Our data suggest that HBx triggers the upregulation of the mRNA and protein levels of B56γ via the inducible AP-1 transactivation.

The ER has a wide variety of functions, such as protein folding and transportation, calcium homeostasis, and lipid synthesis^38^. Many xenobiotics- or viruses-induced ER stress, resulting in accumulation of unfolded or misfolded proteins in ER. Then, unfolded protein response (UPR) is triggered to maintain ER homeostasis. PERK (protein kinase RNA-like endoplasmic reticulum kinase), IRE1α (inositol-requiring enzyme 1α), and ATF6 are the three major UPR branches. The UPR is activated to reduce misfolded proteins, decrease the transcription of mRNAs, and promote autophagy and apoptosis when unfolded proteins are overloaded^38^. ER stress might play an important role in the liver progressive complications caused by HBx^26^. Cho et al.^27^ has reported that HBx activates CREBH through c-Jun (one of AP-1 component) binding to CREBH promoter to enhance its transcription in hepatic cells. Similarly, we found that CREBH, the hepatic ATF6 homolog, was activated by HBx in HBV-infected HepG2-NTCP cells and HBx-expressing cells. Moreover, AP-1 activation, B56γ induction as well as cell apoptosis caused by HBx were reversed by blocking ER stress with either its inhibitor (4-PBA) or siRNA for *HSPA5* or *CREBH*. These data suggest that B56γ is a downstream protein of UPR and might be partially regulated by UPR. Also, CREBH signaling is proved to be associated with AP-1-transactivated *PPP2R5C* upregulation in hepatic cells, leading to B56γ-harbored PP2A activity elevation to mediate HBx-induced apoptosis.

**Fig. 8:**
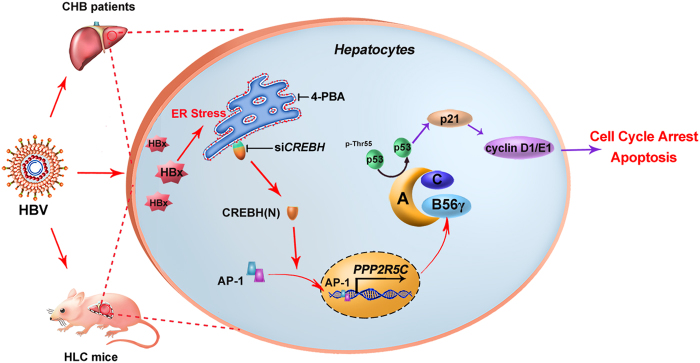
The scheme for molecular mechanisms of B56γ, targeted by HBx, mediates cell cycle arrest and apoptosis of hepatocytes. Among chronic hepatitis B (CHB) patients, livers from HBV-infected primary human hepatocytes (PHHs) in human-liver-chimeric (HLC) mice, HBx-transgenic (Tg) mice, and HBx-expressing hepatic cells, B56γ is induced by HBx through ER stress CREBH signaling and AP-1 transcription regulation. Upregulation of B56γ is proved to promote cell cycle arrest and apoptosis through dephosphorylating p-Thr55-p53 and its downstream p21 pathway in hepatocytes

In conclusion, we have demonstrated that B56γ expression positively correlates with HBx in HBV-infected HLC mice, HBx-Tg mice, HBV-infected and HBx-expressing hepatic cells. B56γ has been proven to promote cell cycle arrest and apoptosis of HBx-expressing hepatic cells both in vivo and in vitro. Mechanistically, as shown in Fig. 8, our study demonstrates that HBx upregulates B56γ expression through AP-1 transactivation, mediated by ER stress CREBH signaling. Then, B56γ-harbored PP2A holoenzyme dephosphorylates p-Thr55-p53 to trigger p53/p21 pathway-dependent cell cycle arrest, resulting in apoptosis and hepatic injury. B56γ, in hope, could be a potential therapeutic target for HBV-related hepatic injury.

## Materials and methods

### GEO data analysis

Gene expression data of CHB cohort were searched from GEO database (http://www.ncbi.nlm.nih.gov/geo/). The relative gene expressions of *PPP2R5C* and *CCNE1* in liver tissues from CHB cohort were analyzed. Pearson’s correlation analysis was used for the relationship between *PPP2R5C* and *CCNE1* mRNA.

### Animal studies

According to method of Azuma et al.^39^, we constructed the HLC mice for HBV infection through transplantation of PHHs into FRG mice. Human hepatocytes, purchased from BD (San Jose, CA, USA), were transplanted into FRG mice after the 2-(2-nitro-4-trifluoro-methylbenzoyl)-1,3-cyclo-hexanedione withdrawal. FRG mice with high human hepatocyte-repopulation were employed to be infected with HBV derived from the supernatant of HepAD38 cells. Persistent infection was established at the 6th week after initial HBV infection^40^. After HBV infection, we isolated the infected hepatocytes for quantitative real-time polymerase chain reaction PCR (qPCR) and IHC analysis. The HBx-Tg mice were constructed via a gene-targeting technique using the pAlb-In-pA-HS4 vector containing full-length *HBX* as a liver-specific transgene as described in a previous study^18^. At 12 months, the HBx-Tg and C57BL/6 WT mice were killed and the livers were excised, fixed, paraffin embedded, and sectioned for analysis. All animal experiments were approved by the Xiamen University Animal Ethics Committee (No. XMULAC20140033) and conducted according to the *Guide for the Care and Use of Laboratory Animals* (National Research Council, US, Eighth edition, 2011).

### Histology and IHC

The tissue sections from the liver of HBV-infected HLC and HBx-Tg mice were deparaffinized in xylene for 20 min and rehydrated with a series of graded ethanol. HE staining was conducted on sections. For TUNEL assay, the apoptotic cells in livers from mice were analyzed using TUNEL Assay Kit from KeyGen BioTech (Nanjing, China) according the manufacturer’s protocol. The bar graph was generated using the data from at least five fields for each sample. For IHC, endogenous peroxidase activity of tissue sections was blocked by a 30 min treatment with 3% hydrogen peroxide in methanol. Sections were incubated with primary antibody in 0.3% bovine serum albumin at 4 °C overnight. They were then incubated for 30 min with a horseradish peroxidase-conjugated secondary antibody and developed with diaminobenzidine and counterstained with hematoxylin. Pictures were taken with an inverted microscope (Nikon, Tokyo, Japan). Brown staining indicated immunoreactivity.

### Cell culture and cell viability assay

The HepG2 cells and HEK-293T cells were maintained in our laboratory. HepG2.2.15, HepaRG, and HepG2-NTCP cells were obtained from the National Institute of Diagnostics and Vaccine Development in Infectious Diseases, Xiamen University. HepG2.2.15 cells integrate two head-to-tail copies of the HBV genome. The human hepatoma HepG2 and HepG2.2.15 cells were cultured in RPMI-1640 medium with 10% fetal bovine serum (FBS). HepG2-Tet-ON-HBx cells were constructed by the methods described in the next section. The HepaRG cells will progressively exhibit the features of functional hepatocytes and bile duct-like cells when treated with dimethyl sulfoxide (DMSO)^41^. We maintained HepaRG cells in Williams E medium with GlutaMAX-I supplemented with 1% penicillin–streptomycin, 4 μg/mL of bovine insulin, and 5 × 10^−5^ mol/L of hydrocortisone hemisuccinate (Sigma, St. Louis, MO, USA). HepaRG cells were seeded at low density for 2 weeks. For differentiation, cells were induced by incubation in culture medium supplemented with 2% DMSO for another 2 weeks. NTCP has been demonstrated to be a functional receptor for HBV^19^. HepG2-NTCP cells were also infected with HBV that derived from the supernatant of HepaAD38 cells. After 4, 6, 8, and 10 days of initial infection, the supernatant of HepG2-NTCP cells were collected for detecting HBsAg and HBeAg using a chemiluminescence immunoassay as previously described^40^. All these cells were cultured in a humidified incubator with 5% CO_2_ at 37 °C. All the reagents for cell culture, except those as otherwise stated, were obtained from Thermo (New Ington, NH, USA). Cell viability assay was used MTS reagent (Promega, Madison, WI, USA) and performed according to manufacturer’s instruction. The optic density was quantified at 490 nm by multifunctional reader system (Multiskan™, Thermo).

### Luciferase reporter gene assay

We performed this assay with pGL3-basic (pGL3b) luciferase reporter plasmids (Promega) containing fragments with different lengths of 5′-flanking region for the *PPP2R5C* promoter. As shown in Table S1, the fragments of the *PPP2R5C* 5′-flanking region include various lengths of promoter, including nucleotides −1701 ~ +136 nt (Dp), −1446 ~ +136 nt (Mp), −694 ~ +136 nt (Pp), and −549 ~ +136 nt (Sp). Transcription start site is named as +1 nt. We also produced AP-1-binding site mutant (−526 C > T) in Sp DNA fragment (*2R5C*-Sp (mut)). These were inserted into the pGL3b vector and the products named pGL3b-*2R5C*-Dp, pGL3b-*2R5C*-Mp, pGL3b-*2R5C*-Pp, pGL3b-*2R5C*-Sp, and pGL3b-*2R5C*-Sp (mut), respectively. When HepG2-Tet-ON-HBx cells were grown up to 80–90% confluence, 50 ng of pRL-TK plasmids and 200 ng of constructed plasmids (pGL3b with one of the truncated *PPP2R5C* promoters) or unmodified pGL3b plasmids were co-transfected into the cells for 4 h using Lipofectamine^®^ 2000 (Thermo). After the cells were treated with or without 1 μg/mL DOX for 24 h, the luciferase intensity was measured using a Dual Luciferase^®^ reporter assay system (Promega). The relative luciferase activity expressed as relative light units was normalized to *Renilla* activity and was relative to pGL3b, which was set as 1.

### Establishment of stable B56γ knockdown and overexpressing cell lines

For the stable B56γ knockdown cell lines, three short hairpin RNAs (shRNAs), targeting *PPP2R5C* (*2R5C*) mRNA at 268 nt-, 417 nt-, and 1416 nt-sites (named as sh268, sh417, and sh1416, respectively), were designed using the Genetic Perturbation Platform (http://www.broadinstitute.org/rnai/public/). Each of the three sh*2R5C* sequences was ligated into the pLKO.1 lentiviral vector to construct the pLKO.1-sh*2R5C* recombinant plasmids, and sh*GFP* was also introduced into the vector, as a control targeting to *GFP* encoding sequence, for a total of four shRNA-containing plasmids. For B56γ-overexpressing cells, the *PPP2R5C* encoding sequence was incorporated into retroviral vector pBabe-puro to construct the pBabe-*2R5C* recombinant plasmids (pB-*2R5C*). All the recombinant viruses infected HepG2 cells. The detailed protocols were described in our previous study^42^. All the polynucleotides, synthesized by Invitrogen (Shanghai, China), are shown in Table S2. These constructions were verified by DNA sequencing, qPCR for *PPP2R5C* mRNA level, and western blot analysis for B56γ protein level. Based on the successful construction, these cells were named as HepG2-sh*2R5C*, HepG2-sh*GFP*, HepG2-*2R5C*, and HepG2-pB, respectively.

### Cell transient transfection

For transient overexpression, an *HBX* DNA fragment was inserted into the pEGFP plasmids; the resulting plasmids were named as pEGFP-HBX. HepaRG cells were transfected with either pEGFP or pEGFP-*HBX* plasmids using Lipofectamine^®^ 2000 according to the manufacturer’s direction. For transient knockdown, cells were transfected with 50 nmol/L annealed double-stranded siRNA for *PPP2R5C* (si*2R5C*), *HSPA5* (si*HSPA5*), *CREBH* (si*CREBH*) (Ribobio, Guangzhou, China) using Lipofectamine^®^2000 according to the manufacturer’s mannual. siNC stands for siRNA negative control. After 24–72 h, cells were analyzed for their suppression efficiency using qPCR and western blot and used in other experiments. All experiments were repeated at least three times independently.

### Establishment of Tet-ON-HBx inducible expression cells

A Tet-ON^®^3G Inducible Expression System was purchased from BD containing pTRE3G-ZsGreen and pCMV-Tet3G vectors. The primers for plasmids construction are listed in Table S2. The HepG2-Tet-ON-HBx cells were constructed according to the manufacturer’s protocol. In brief, HepG2 cells were first transduced with the pCMV-Tet3G lentivirus and were screened with G418 (Roche Diagnostics, Indianapolis, IN, USA) at 500 µg/mL for 14 days. Then, the Tet-ON cells were transduced with the pTRE3G-HBx-ZsGreen lentivirus. After selection with 0.6 µg/mL puromycin, the doubly transduced cells were treated with 0.5 or 1 μg/mL DOX for 24 h to induce HBx expression.

### RT-PCR and qPCR

Total RNA was isolated from the tissues or cells using TRIzol reagent (TaKaRa, Osaka, Japan). RNA was reverse transcribed to cDNA by reverse transcriptase (TaKaRa) according to the manufacturer’s protocol. Reverse transcription-polymerase chain reaction was performed using specific primers. The transcription level of target genes was visualized using agarose gel electrophoresis. Each reaction was repeated independently at least three times. QPCR was conducted with SYBR^®^ Premix ExTaq^TM^ II Kit (TaKaRa) using a CFX96 Touch^TM^ Detection System (Bio-Rad, Hercules, CA, USA). Cycling conditions were 95 °C for 30 s, 40 cycles of 95 °C for 5 s, and 60 °C for 34 s. All primers are described in Table S2. The expression of the target genes were evaluated using the 2^-△△Ct^ relative quantification method, normalized to their corresponding reference gene (*ACTB* for human genes and *Gapdh* for mouse genes).

### Western blot

Extractions of proteins from tissues, whole cells, and cytoplasmic and nuclear fractions, and western blot were performed as previously described^42^. The detailed information about the antibodies was presented in Table S3. The HBx antibody was produced as described in our previous study^43^. The primary antibodies for anti-p53, -p21, -cyclin D1, the ER stress antibody sample kit (dilution 1:1000), and the biotin-labeled secondary antibodies (anti-rabbit IgG and anti-mouse IgG) (dilution 1:10000) were purchased from CST (Danvers, MA, USA). Anti-p-p53 (Thr55) and -CREBH antibodies were purchased from Thermo (dilution 1:500). Anti-c-Jun and -B56γ antibodies were obtained from Abcam (Cambridge, MA, USA; dilution 1:1000). The antibodies against caspase 3, GAPDH, and Histone H3 were obtained from Beyotime (Shanghai, China; dilution 1:1000). The antibodies against cyclin E1 and CK-8 were purchased from Ruiying Biological (Suzhou, China; dilution 1:1000).

### Protein phosphatase activity assay

Cells were lysed according to the manufacturer’s instruction of the Serine/Threonine Phosphatase Assay Kit (Promega) and our previous method^42^. The cell lysates in phosphatase storage buffer were centrifuged at 1 × 10^5^ *g* at 4 °C for 1 h. Phosphatase storage buffer contained 2 mmol/L EGTA, 5 mmol/L EDTA, 0.5 mmol/L PMSF, 150 mmol/L NaCl, 1% Triton X-100, 50 mmol/L Tris-HCl (pH 7.4), and 0.5% protease inhibitor cocktail. Following the instructions, equal volumes of cell lysates were added into the reaction buffer (50 μL total), and incubated at 37 °C for 1 h. The reaction was stopped by adding 50 μL of molybdate dye/additive mixture and incubated for 30 min. The optical density at 600 nm of the samples was read using a Multiskan^TM^ FC microplate photometer (Thermo). This assay was performed in three independent experiments.

### EMSA

We performed a search for potential transcription factor-binding sites in the promoter of *PPP2R5C* using a gene-regulation prediction online tool (http://gene-regulation.com/pub/databases.html)^44^. The names, locations, and transcription factors binding to each DNA fragment were given in Table S1. To make probes for these sites, single-stranded oligonucleotides and their antisense oligonucleotides (Table S4) were synthesized by Sangon (Shanghai, China). Biotinylated or unlabeled oligonucleotides with their complementary oligonucleotides were annealed to make double-stranded biotin-labeled hot probes or unlabeled cold probes. Nuclear components were extracted from HepG2-Tet-ON-HBx cells using a Nuclear Protein Separation Kit (Beyotime). EMSA was performed according to the protocol for the LightShift^TM^ Chemiluminescent EMSA Kit (Thermo). The nuclear components and each probe were hybridized for 20 min, followed by loading into a 6% polyacrylamide gel, electrophoresis, and transfer to a nylon membrane (Thermo). The blots were treated with streptavidin-horseradish peroxidase conjugate and chemiluminescent substrate and the location of the probes detected using X-ray film in the dark.

### Immunofluorescence

The tissue sections from the livers of HBV-infected HLC mice were deparaffinized in xylene for 20 min and rehydrated with a series of graded ethanol. HepG2-Tet-ON-HBx cells were grown on coverslips in six-well plates at 37 °C for 24 h and fixed in 4% paraformaldehyde (pH 7.4) for 15 min. After washing twice with PBS, the slices of tissues or cells were incubated in PBS plus 0.3% Triton X-100 for 10 min. The slices were then blocked with 3% FBS for 30 min and incubated with primary antibodies (anti-HBx, -B56γ, -c-Jun or -CREBH, dilution 1:200) at 4 °C overnight. After washing three times with ice-cold PBS, the slices were incubated with Alexa Fluor^®^594 goat anti-rabbit IgG (Thermo; dilution 1:400) and Alexa Fluor^®^488 goat anti-mouse IgG (Beyotime; dilution 1:400) at room temperature for 1 h in the dark. After extensive washing with ice-cold PBS, the nuclei were counterstained with 4′,6-diamidino-2-phenylindole (Beyotime). The samples were photographed using a confocal microscope (Zeiss, Oberkochen, Germany). Pearson’s correlation for the colocalization of B56γ and HBx proteins was caculated with GraphPad software.

### Flow cytometry

The cell cycle analysis was described as our previous study^42^. In brief, after HepG2-Tet-ON-HBx transfected with si*2R5C* or siNC for 8 h, the cells were recovered for another 24 h with changing fresh medium and then induced by DOX. HepG2-pB and HepG2-*2R5C* cells were transfected with pcDNA3.1-*HBX* or pcDNA3.1 plasmids together with si*2R5C* or siNC for 8 h, and then recovered for another 24 h. These cells were fixed in 70% cold ethanol, then digested with RNase, and finally stained with propidium iodide (PI). FCM (Beckman, Brea, CA, USA) was applied to measure DNA content. For apoptosis assay, a commercial Annexin-V-FITC Apoptosis Kit (Beyotime) was used according to the manufacturer’s instruction. In brief, 3 × 10^5^ cells were gently resuspended and incubated for 10 min at room temperature in the dark with Annexin-V-FITC antibody. Samples were then stained with PI (50 μg/mL). For each sample, at least 10000 events were collected. These FCM data were handled with FlowJo v.7.6.5 (FlowJo, Ashland, OR, USA). The experiments were repeated at least three times.

### Statistics

Statistical analyses used the statistical package for social sciences (SPSS) version 16.0 (SPSS, Chicago, IL, USA). All data are shown as the mean ± standard deviation. Statistical analyses were performed using one-way analysis of variance and Student’s *t* test. Pearson’s correlation analysis was conducted for variables correlation. *P* *<* 0.05 was considered to be statistically significant.

## Electronic supplementary material

CDDIS-18-0357R-Supplementary files

## References

[1] World Health Organization, *Global hepatitis report*. (2017) http://www.who.int/mediacentre/factsheets/fs204/en/.

[2] Davis GL (2008). Hepatocellular carcinoma: management of an increasingly common problem. Proc. (Bayl. Univ. Med. Cent.)..

[3] Huang JF (2016). EVI1 promotes cell proliferation in HBx-induced hepatocarcinogenesis as a critical transcription factor regulating lncRNAs. Oncotarget.

[4] Seeger C, Mason WS (2015). Molecular biology of hepatitis B virus infection. Virology.

[5] Murakami S (2001). Hepatitis B virus X protein: a multifunctional viral regulator. J. Gastroenterol..

[6] Riviere L, Ducroux A, Buendia MA (2014). The oncogenic role of hepatitis B virus. Recent Results Cancer Res..

[7] Levrero M, Zucman-Rossi J (2016). Mechanisms of HBV-induced hepatocellular carcinoma. J. Hepatol..

[8] McConnell JL, Wadzinski BE (2009). Targeting protein serine/threonine phosphatases for drug development. Mol. Pharmacol..

[9] Strack S (2002). Protein phosphatase 2A holoenzyme assembly: identification of contacts between B-family regulatory and scaffolding A subunits. J. Biol. Chem..

[10] Lechward K, Awotunde OS, Swiatek W, Muszynska G (2001). Protein phosphatase 2A: variety of forms and diversity of functions. Acta Biochim. Pol..

[11] McCright B, Rivers AM, Audlin S, Virshup DM (1996). The B56 family of protein phosphatase 2A (PP2A) regulatory subunits encodes differentiation-induced phosphoproteins that target PP2A to both nucleus and cytoplasm. J. Biol. Chem..

[12] Shouse GP, Nobumori Y, Panowicz MJ, Liu X (2011). ATM-mediated phosphorylation activates the tumor-suppressive function of B56γ-PP2A. Oncogene.

[13] Li HH, Cai X, Shouse GP, Piluso LG, Liu X (2007). A specific PP2A regulatory subunit, B56γ, mediates DNA damage-induced dephosphorylation of p53 at Thr55. EMBO J..

[14] Chen W (2004). Identification of specific PP2A complexes involved in human cell transformation. Cancer Cell.

[15] Guergnon J (2011). PP2A targeting by viral proteins: a widespread biological strategy from DNA/RNA tumor viruses to HIV-1. Biochim. Biophys. Acta.

[16] Chen Y (2007). Structural and biochemical insights into the regulation of protein phosphatase 2A by small t antigen of SV40. Nat. Struct. Mol. Biol..

[17] Gong SJ (2016). Upregulation of PP2Ac predicts poor prognosis and contributes to aggressiveness in hepatocellular carcinoma. Cancer Biol. Ther..

[18] Wu BK (2006). Blocking of G1/S transition and cell death in the regenerating liver of Hepatitis B virus X protein transgenic mice. Biochem. Biophys. Res. Commun..

[19] Yan H (2012). Sodium taurocholate cotransporting polypeptide is a functional receptor for human hepatitis B and D virus. eLife.

[20] Sells MA, Chen ML, Acs G (1987). Production of hepatitis B virus particles in HepG2 cells transfected with cloned hepatitis B virus DNA. Proc. Natl. Acad. Sci. USA.

[21] Wang T (2011). Hepatitis B virus induces G1 phase arrest by regulating cell cycle genes in HepG2.2.15 cells. Virol. J..

[22] Lucifora J (2011). Hepatitis B virus X protein is essential to initiate and maintain virus replication after infection. J. Hepatol..

[23] Qiao L (2013). SREBP-1α activation by HBx and the effect on hepatitis B virus enhancer II/core promoter. Biochem. Biophys. Res. Commun..

[24] Benn J, Su F, Doria M, Schneider RJ (1996). Hepatitis B virus HBx protein induces transcription factor AP-1 by activation of extracellular signal-regulated and c-Jun N-terminal mitogen-activated protein kinases. J. Virol..

[25] Kataoka K, Noda M, Nishizawa M (1994). Maf nuclear oncoprotein recognizes sequences related to an AP-1 site and forms heterodimers with both Fos and Jun. Mol. Cell Biol..

[26] Li B (2007). Hepatitis B virus X protein (HBx) activates ATF6 and IRE1-XBP1 pathways of unfolded protein response. Virus Res..

[27] Cho HK (2015). HBx induces the proliferation of hepatocellular carcinoma cells via AP1 over-expressed as a result of ER stress. Biochem. J..

[28] Chen L (2014). Cab45S inhibits the ER stress-induced IRE1-JNK pathway and apoptosis via GRP78/BiP. Cell Death Dis..

[29] Li T (2012). Tumor suppression in the absence of p53-mediated cell-cycle arrest, apoptosis, and senescence. Cell.

[30] Pim D, Massimi P, Dilworth SM, Banks L (2005). Activation of the protein kinase B pathway by the HPV-16 E7 oncoprotein occurs through a mechanism involving interaction with PP2A. Oncogene.

[31] Kim N, Kukkonen S, Gupta S, Aldovini A (2010). Association of Tat with promoters of PTEN and PP2A subunits is key to transcriptional activation of apoptotic pathways in HIV-infected CD4^+^ T cells. PLoS. Pathog..

[32] Kachnic LA (1999). The ability of p53 to activate downstream genes p21^(WAF1/cip1^) and MDM2, and cell cycle arrest following DNA damage is delayed and attenuated in scid cells deficient in the DNA-dependent protein kinase. J. Biol. Chem..

[33] Kumar R, Gururaj AE, Barnes CJ (2006). p21-activated kinases in cancer. Nat. Rev. Cancer.

[34] Chen Y (2013). Differential gene expression profiles of *PPP2R5C*-siRNA-treated malignant T cells. DNA Cell Biol..

[35] Klein A (2003). HBX causes cyclin D1 overexpression and development of breast cancer in transgenic animals that are heterozygous for p53. Oncogene.

[36] Luo MX (2015). Autophagy mediates HBx-induced nuclear factor-κB activation and release of IL-6, IL-8, and CXCL2 in hepatocytes. J. Cell Physiol..

[37] Tanaka Y (2006). The hepatitis B virus X protein enhances AP-1 activation through interaction with Jab1. Oncogene.

[38] Bravo R (2013). Endoplasmic reticulum and the unfolded protein response: dynamics and metabolic integration. Int. Rev. Cell Mol. Biol..

[39] Azuma H (2007). Robust expansion of human hepatocytes in Fah^-/-/^Rag2^-/-/^Il2rg^-/-^ mice. Nat. Biotechnol..

[40] Zhang TY (2016). Prolonged suppression of HBV in mice by a novel antibody that targets a unique epitope on hepatitis B surface antigen. Gut.

[41] Cerec V (2007). Transdifferentiation of hepatocyte-like cells from the human hepatoma HepaRG cell line through bipotent progenitor. Hepatology.

[42] Zhuang Q (2016). Protein phosphatase 2A-B55δ enhances chemotherapy sensitivity of human hepatocellular carcinoma under the regulation of microRNA-133b. J. Exp. Clin. Cancer Res..

[43] Geng X (2012). Hepatitis B virus X protein targets Bcl-2 proteins to increase intracellular calcium, required for virus replication and cell death induction. Proc. Natl. Acad. Sci. USA.

[44] Matys V (2006). TRANSFAC and its module TRANSCompel: transcriptional gene regulation in eukaryotes. Nucleic Acids Res..

