# Supplementary material for: ER stress regulating protein phosphatase 2A-B56γ, targeted by hepatitis B virus X protein, induces cell cycle arrest and apoptosis of hepatocytes
Source: Cell Death Dis. 2018 Jul 9;9(7):762. doi: 10.1038/s41419-018-0787-3 (PMC6037732; doi:10.1038/s41419-018-0787-3)
Supplement: Supplementary file 1 — CDDIS-18-0357R-Supplementary files [file 41419_2018_787_MOESM1_ESM.doc]

**Supplementary Files**

**Table S1. Potential transcription factor binding sites on the *PPP2R5C* promoter.**

| **5’-flanking region** | **Name** | [**Transcription**](../../../../C:/Users/Administrator/AppData/Local/Yodao/DeskDict/frame/20150803170523/javascript:void(0)%3B) [**factor**](../../../../C:/Users/Administrator/AppData/Local/Yodao/DeskDict/frame/20150803170523/javascript:void(0)%3B)**s** |
| --- | --- | --- |
| -1701 ~ +136 nt | Dp | NF-κB, NF-1, SP1, AP-1, Oct-1, CPC1, NF-E2, p40x, CP1 |
| -1446 ~ +136 nt | Mp | SP1, AP-1, Oct-1, CPC1, NF-E2, p40x, CP1, NF-κB |
| -694 ~ +136 nt | Pp | CPC1, AP-1, NF-E2, p40x, CP1, NF-κB |
| -549 ~ +136 nt | Sp | AP-1, NF-E2, p40x, CP1, NF-κB |

Abbreviations: AP-1, activator protein-1; CP1, CCAAT transcription factor; CPC1, cross pathway control-1; Dp, distant promoter; Mp, middle promoter; NF-1, nuclear factor-1; NF-E2, nuclear factor, erythroid 2; NF-κB, nuclear factor-kappa B; Oct-1, octamer binding transcription factor-1; p40x, the gene product of frame IV (x-lor); Pp, proximal promoter; Sp, short promoter; SP1, specificity protein 1.

**Table S2. Primers and their sequences used in this study.**

| **Primer name** | **Sequence (5' - 3')** | **Usage** |
| --- | --- | --- |
| *2R5C*-sh268FP | CCGGTCCAGAAGTTACGTCAGTGTTCTCGAGAACACTGACGTAACTTCTGGATTTTTG | sh*2R5C* |
| *2R5C*-sh268RP | AATTCAAAAATCCAGAAGTTACGTCAGTGTTCTCGAGAACACTGACGTAACTTCTGGA | sh*2R5C* |
| *2R5C*-sh417FP | CCGGCCAGAAGTAGTCCATATGTTTCTCGAGAAACATATGGACTACTTCTGGTTTTTG | sh*2R5C* |
| *2R5C*-sh417RP | AATTCAAAAACCAGAAGTAGTCCATATGTTTCTCGAGAAACATATGGACTACTTCTGG | sh*2R5C* |
| 2R5C-sh1416FP | CCGGCGGGAAGAAGCATGGGTTAAACTCGAGTTTAACCCATGCTTCTTCCCGTTTTTG' | sh*2R5C* |
| *2R5C*-sh1416RP | AATTCAAAAACGGGAAGAAGCATGGGTTAAACTCGAGTTTAACCCATGCTTCTTCCCG | sh*2R5C* |
| HBx+205qRTFP | TGTGCACTTCGCTTCA | qPCR |
| HBx+465qRTRP | TTAGGCAGAGGTGAAAAAGTT | qPCR |
| LvTet-ON-*HBX*+1MF | GAATACGCGTATGGCTGCTAGGYTGTRCTG | Tet-ON-HBx |
| LvTet-ON-*HBX*+465ER | GAATGAATTCTTAGGCAGAGGTGAAAAAGTT | Tet-ON-HBx |
| pLVX-TRE3G-ZsGreen1-MluI-*HBX*+1FP | gcccccgggacgcgtATGGCTGCTAGGTTGTACTG | Tet-ON-HBx |
| pLVX-TRE3G-ZsGreen1-EcoRI-*HBX*+462RP | ctacccggtagaattcTTAGGCAGAGGTGAAAAAGT | Tet-ON-HBx |
| pBabe-*HBX*+ECOR+1FP | CAAGGAATTCATGGCTGCTAGGTTGTAC | Stable HBx overexpression |
| pBabe-*HBX*+SaL+465RP | CAAGGTCGACTTAGGCAGAGGTGAAAAAGT | Stable HBx overexpression |
| *ALB*+1346qRTFP | TACAAATTCCAGAATGCGCT | qPCR |
| *ALB*+1518qRTRP | TTCAGGACCACGGATAGAT | qPCR |
| *HNF4A*+392qRTFP | ACATGTACTCCTGCAGAT | qPCR |
| *HNF4A*+617qRTRP | GAGGTGATCTGTCGGGA | qPCR |
| *NTCP*+801qRTFP | ATTGCCACCTCCTCCCTGAT | qPCR |
| *NTCP*+1071qRTRP | CAGTCTTGAATTTCTCATAGCACC | qPCR |
| *PPP2R5C-*qRTFP | CAAAGCCAATCCCCAGTAC | qPCR |
| *PPP2R5C*-qRTRP | TCGGATCTTTCTGTGCCTGA | qPCR |
| *Ppp2r5c*-RTFP | CAAAGCGAATCCCCAGTAT | RT-PCR |
| *Ppp2r5c*-RTRP | TCAGCTCCTTCTGTGCCTGA | RT-PCR |
| *ACTB*-qRTFP | CACCAGGGCGTGATGGT | qPCR |
| *ACTB*-qRTRP | CTCAAACATGATCTGGGTCAT | qPCR |
| *Gadph*-RTFP | TTGATGGCAACAATCTCCAC | RT-PCR |
| *Gapdh*-RTRP | CGTCCCGTAGACAAAATGGT | RT-PCR |

Abbreviations: *2R5C*, *PPP2R5C*; *ALB*, albumin gene; FP, forward primer; *Gapdh*, mouse glyceraldehyde 3-phosphate dehydrogenase gene; *HBX*, gene encoding hepatitis B virus X protein; *HNF4A*, hepatocyte nuclear factor 4 alpha gene; Lv, lentivirus; *NTCP*, sodium taurocholate cotransporting polypeptide gene; qPCR, quantitative real-time polymerase chain reaction; RP, reverse primer; RT-PCR, reverse transcription-polymerase chain reaction; shRNA, short hairpin RNA.

**Table S3. Detailed information of antibodies used in this study.**

| **Antibody Name** | **Manufacturer** | **Catalog number** |
| --- | --- | --- |
| Anti-HBx | mAb(16F9) Produced by our lab and reported in previous study. | Ref: Ge et al. Proc Natl Acad Sci U S A 2012;109: 18471-18476. |
| Anti-HBsAg | Produced by our lab and reported in previous study. | Ref: Zhang et al. Gut 2016;65:658-671. |
| Anti-HBcAg | DAKO | B058601 |
| Anti-CREBH | Thermo | PA5-31708 |
| Anti-B56γ | Thermo | 39-3600 |
| Alexa Fluor®594 goat anti-rabbit IgG | Thermo | 35560 |
| Anti-p-p53 (Thr55) | Abcam | ab183546 |
| Anti-p53 | CST | 9919S |
| Anti-p21 | CST | 2947 |
| Anti-c-Jun | CST | 9165S |
| Anti-cyclin D1 | CST | 2978 |
| ER stress antibody sample kit | CST | 9956S |
| Anti-rabbit HRP-conjugated IgG secondary antibody | CST | 31460 |
| Anti-mouse HRP-conjugated IgG secondary antibody | CST | 31430 |
| Anti-GAPDH | Beyotime | AG019 |
| Anti-Histone H3 | Beyotime | AH433 |
| Alexa Fluor®488 goat anti-mouse IgG | Beyotime | A0423 |
| Anti-cyclin E1 | Ruiying Biological | RLT1178 |
| Anti-CK-8 | Ruiying Biological | RLM3055 |

Abbreviations: Anti-, antibody; CK-8, cytokeratin-8; CREBH, cAMP-responsive element-binding protein, hepatocyte specific; ER, endoplasmic reticulum; GAPDH, glyceraldehyde 3-phosphate dehydrogenase; HBcAg, hepatitis B core antigen; HBsAg, hepatitis B surface antigen; HBx, hepatitis B virus X protein; HRP, horseradish peroxidase..

**Table S4. EMSA probes used in this study.**

| **Probe name** | **Sequence** |
| --- | --- |
| NF-κB-5BHprobeS | 5´Biotin-AGTTGAGGGGACTTTCCCAGGC-3´ |
| NF-κB-5BHprobeAS | 5´Biotin-GCCTGGGAAAGTCCCCTCAACT-3' |
| NF-κB-CprobeS | 5´-AGTTGAGGGGACTTTCCCAGGC-3´ |
| NF-κB-probeAS | 5´-GCCTGGGAAAGTCCCCTCAACT-3' |
| AP-1(cJun)-5BHprobeS | 5´Biotin-CGCTTGATGAGTCAGCCGGAA-3´ |
| AP-1(cJun)-5BHprobeAS | 5´Biotin-TTCCGGCTGACTCATCAAGCG-3' |
| AP-1(cJun)-CprobeS | 5´-CGCTTGATGAGTCAGCCGGAA-3´ |
| AP-1(cJun)-CprobeAS | 5´-TTCCGGCTGACTCATCAAGCG-3' |
| *2R5C*p-5BHprobeS | 5´Biotin-GGATCATAAGTTAATAACTCATCCTATTGGTAAGGGAAGT-3' |
| *2R5C*p-5BHprobeAS | 5´Biotin-ACTTCCCTTACCAATAGGATGAGTTATTAACTTATGATCC-3' |
| *2R5C*p-CprobeS | 5´-GGATCATAAGTTAATAACTCATCCTATTGGTAAGGGAAGT-3' |
| *2R5C*p-CprobeAS | 5'-ACTTCCCTTACCAATAGGATGAGTTATTAACTTATGATCC-3' |

Abbreviations: *2R5C*, *PPP2R5C*; AP-1, activator protein-1; BHprobe, Biotin-labeled hot probe; Cprobe, unlabeled cold probe; EMSA, electrophoretic mobility shift assay; NF-κB, nuclear factor-kappa B.


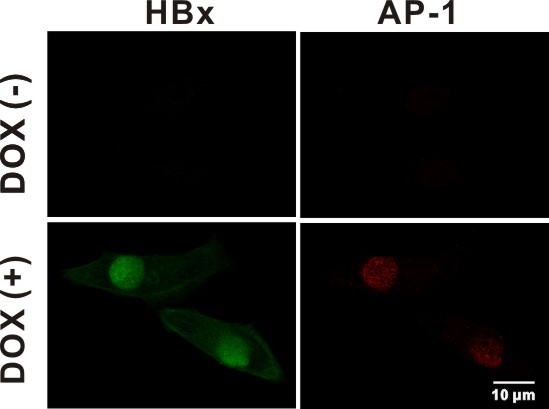


**Figure S1. AP-1 transactivation in HepG2-Tet-ON-HBx cells with HBx expression.** After DOX (1 μg/mL) induction for 24 h, immunofluorescence analyses for HBx and AP-1 subunit c-Jun were conducted and imaged with a confocal microscopy. Left panel, HBx expression; right panel, AP-1 subunit c-Jun nucleus translocation. Scale bar = 10 μm.


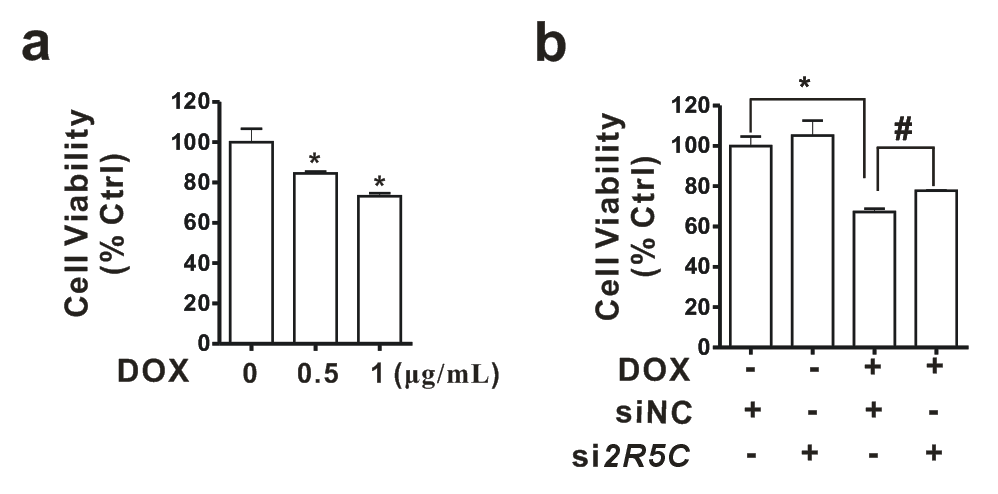


**Figure S2. Knockdown of B56γ rescues the decrease of cell viability caused by HBx in hepatic cells.** (**a**) Cell viability in HepG2-Tet-ON-HBx cells upon DOX (0.5 and 1 μg/mL) induction was detected using MTS assay. * *P* < 0.05 as compared to control. (**b**) Cell viability in HepG2-Tet-ON-HBx cells upon DOX (1 μg/mL) induction following si*2R5C* or siNC transfection was detected using MTS assay. * *P* < 0.05 as compared to cells transfected siNC without DOX induction. ^#^ *P* < 0.05 as compared to cells with siNC transfection and DOX induction.


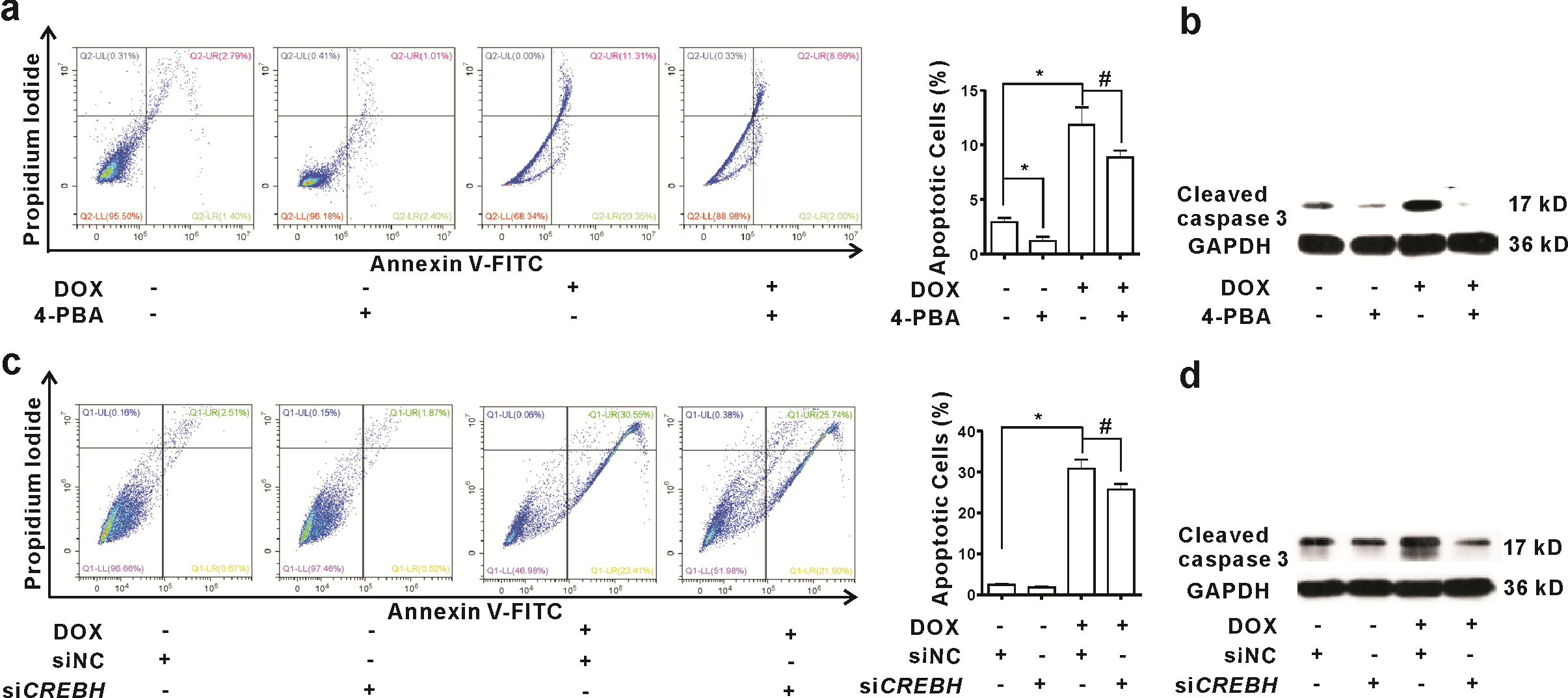


**Figure S3. Blocking ER stress attenuates HBx-triggered apopotosis in hepatocytes.** (**a-b**) HepG2-Tet-ON-HBx cells were pretreated with 4-PBA (5 mM) for 5 h and then induced by DOX (1 μg/mL). (**a**) The apoptotic cells were measured with Annexin V-PI Kit using FCM (left panel, FCM images; right panel, bar graphs). * *P* < 0.05 as compared to control. ^#^ *P* < 0.05 as compared to cells only induced by DOX. (**b**) Cleaved caspase 3 protein was detected using Western blot. (**c-d**) Prior to DOX (1 μg/mL) induction, HepG2-Tet-ON-HBx cells were transfected with si*CREBH* or siNC. (**c**) The apoptotic cells were measured with Annexin V-PI Kit using FCM (left panel, FCM images; right panel, bar graphs), * *P* < 0.05 as compared with siNC. ^#^ *P* < 0.05 as compared with cells with siNC transfection and DOX induction. (**d**) Western blot for cleaved caspase 3 protein.
